# Hyper-power series and generalized real analytic functions

**DOI:** 10.1007/s00605-023-01849-8

**Published:** 2023-03-29

**Authors:** Diksha Tiwari, Akbarali Mukhammadiev, Paolo Giordano

**Affiliations:** Faculty of Mathematics, University of Vienna, Wien, Austria

**Keywords:** Colombeau generalized numbers, Non-Archimedean rings, Generalized functions, 46F-XX, 46F30, 26E3

## Abstract

This article is a natural continuation of the paper Tiwari, D., Giordano, P., *Hyperseries in the non-Archimedean ring of Colombeau generalized numbers* in this journal. We study one variable hyper-power series by analyzing the notion of radius of convergence and proving classical results such as algebraic operations, composition and reciprocal of hyper-power series. We then define and study one variable generalized real analytic functions, considering their derivation, integration, a suitable formulation of the identity theorem and the characterization by local uniform upper bounds of derivatives. On the contrary with respect to the classical use of series in the theory of Colombeau real analytic functions, we can recover several classical examples in a non-infinitesimal set of convergence. The notion of generalized real analytic function reveals to be less rigid both with respect to the classical one and to Colombeau theory, e.g. including classical non-analytic smooth functions with flat points and several distributions such as the Dirac delta. On the other hand, each Colombeau real analytic function is also a generalized real analytic function.

## Introduction

In this article, the study of hyperseries in the non-Archimedean ring of Colombeau generalized numbers (CGN), as carried out in [25], is applied to the corresponding notion of hyper-power series. As we will see, this yields results which are more closely related to classical ones, such as, e.g. the equality  that holds for all  where the exponential is moderate, i.e. if $|x|\le \log \left( \textrm{d}{\rho }^{-R}\right)$ for some $R\in \mathbb {N}$. On the other hand, we will see that classical smooth but non-analytic functions, e.g. smooth functions with flat points, and Schwartz distributions like the Dirac delta, are now included in the related notion of *generalized real analytic function* (GRAF). This implies that necessarily we cannot have a trivial generalization of the identity theorem (see e.g. [20, Corollary 1.2.6, 1.2.7]) but, on the contrary, only a suitable sufficient condition (see Theorem 40 below). The notion of generalized real analytic function hence reveals to be less rigid than the classical concept, by including a large family of non-trivial generalized functions (e.g. Dirac delta $\delta$, Heaviside function *H*, but also powers $\delta ^{k}$, $k\in \mathbb {N}$, and compositions $\delta \circ \delta$, $\delta ^{k}\circ H^{h}$, $H^{h}\circ \delta ^{k}$, etc., for *h*, $k\in \mathbb {N}$.

Conversely, GRAF preserve a lot of classical results: they can be thought of as infinitely long polynomials , with uniquely determined coefficient $a_{n}=\frac{f^{(n)}(c)}{n!}$, they can be added, multiplied, composed, differentiated, integrated term by term, are closed with respect to inverse function, etc. This lays the foundation for a potential interesting generalization of the Cauchy-Kowalevski theorem which is able to include many non-analytic (but generalized real analytic) generalized functions.

Concerning the theory of analytic Colombeau generalized functions, as developed in [23] for the real case and in [1, 2, 5–7, 18, 22, 26] for the complex one, it is worth to mention that several properties have been proved in both cases: closure with respect to composition, integration over homotopic paths, Cauchy integral theorem, existence of analytic representatives, identity theorem on a set of positive Lebesgue measure, etc. (cf. [23, 26] and references therein). On the other hand, even if in [26] it is also proved that each complex analytic Colombeau generalized functions can be written as a Taylor series, necessarily this result holds only in an infinitesimal neighborhood of each point. The impossibility to extend this property to a finite neighborhood is a general drawback of the use of ordinary series in a (Cauchy complete) non-Archimedean framework instead of hyperseries, as explained in details in [25].

We refer to [21] for basic notions such as the ring of Robinson-Colombeau, subpoints, hypernatural numbers, supremum, infimum and hyperlimits, and [25] for the notion of hyperseries as well as their notations and properties. Once again, the ideas presented in the present article can be useful to explore similar ideas in other non-Archimedean settings, such as [3, 4, 15–17, 19, 24].

## Hyper-power series and its basic properties

### Definition of hyper-power series

In the entire paper, $\rho$ and $\sigma$ are two arbitrary gauges; only when it will be needed, we will assume a relation between them, such as $\sigma \le \rho ^{*}$ or $\sigma \ge \rho ^{*}$ (see [25]).

A power series of *real numbers* is usually simply defined as “a series of the form $\sum _{n\in \mathbb {N}}a_{n}(x-c)^{n}$, where *x*, *c*, $a_{n}\in \mathbb {R}$ for all $n\in \mathbb {N}$”. Actually, this (informal) definition does not state explicitly whether a non-convergent series is included or not. However, unlike the real case where finite sums can always be considered, this does not hold for hyperfinite sums in the ring , see [25]. For this reason, we consider the -module  of sequences for hyperseries exactly as the space where we can consider hyperfinite sums regardless of convergence. This is the idea to define the space  of formal hyper-power series (HPS):

#### Definition 1

Let *x*, . We say  if and only if there exist $\left( a_{n\varepsilon }\right) _{n,\varepsilon }\in \mathbb {R}^{\mathbb {N}\times I}$ and representatives $[x_{\varepsilon }]=x$, $[c_{\varepsilon }]=c$ such that2.1

For the notation $\left[ -\right] _{\text {s}}$, see [25, Defi 1]. Elements of  are called *formal* HPS because here we are not considering their convergence. In other words, a formal HPS is a hyper series (i.e. an equivalence class  in the space of sequences for hyperseries) of the form $\left[ a_{n\varepsilon }\cdot \left( x_{\varepsilon }-c_{\varepsilon }\right) ^{n}\right] _{{\textrm{s}}}$.

#### Remark 2

i We explicitly note that $x-c$ is not an indeterminate, like in the case of formal power series $\mathbb {R}\llbracket x\rrbracket$, but a generalized number of . For example, in Lemma 10 below, we will prove that if $x-c=y-d$, then .
ii On the contrary with respect to the case of real numbers, being a formal HPS, i.e. an element of , depends on the interplay of the two gauges $\rho$ and $\sigma$: take e.g. $a_{n}=\frac{1}{n^{2}}$ and $x-c=2$, so that for all  we have $\sum _{n=1}^{N}a_{n}(x-c)^{n}\ge \sum _{n=0}^{N}\frac{1}{n}\sim \log (N)$. Therefore, taking e.g. $\sigma _{\varepsilon }=\exp \left( -\exp \left( \frac{1}{\rho _{\varepsilon }}\right) \right)$ and $N_{\varepsilon }:=\text {int}\left( \sigma _{\varepsilon }\right)$, we have that $\left( \log N_{\varepsilon }\right) \notin \mathbb {R}_{\rho }$ and hence we cannot even consider hyperfinite sums of this form. Informally stated, for this gauge $\sigma$, we have that  is *not* a formal HPS, i.e. even before considering its convergence or not, we cannot compute $\sigma$-hyperfinite sums and get a number in .
iii In [25], we proved that if *x* is finite, then  is a formal HPS for all gauges $\rho$, $\sigma$. In Sect. 14, we will prove that ; on the other hand, we will also see that if $x\le \log \left( \textrm{d}\rho ^{-N}\right)$ and $\textrm{d}\sigma ^{Q}\le \textrm{d}\rho ^{N}$ for some $Q\in \mathbb {N}$, then  is a formal HPS.

The previous Definition 1 sets immediate problems concerning independence of representatives: every time we start from , for all $n\in \mathbb {N}$, $[x_{\varepsilon }]=x$, $[c_{\varepsilon }]=c$ and we have that , we can consider whether the corresponding formal HPS  converges or not. On the other hand, we also have to prove that it is well-defined, i.e. that taking different representatives $\left[ {\bar{a}}_{n\varepsilon }\right] =\left[ a_{n\varepsilon }\right]$, $[{\bar{x}}_{\varepsilon }]=x$, $[{\bar{c}}_{\varepsilon }]=c$, we have $\left[ a_{n\varepsilon }\cdot \left( x_{\varepsilon }-c_{\varepsilon }\right) ^{n}\right] _{{\textrm{s}}}=\left[ {\bar{a}}_{n\varepsilon }\cdot \left( {\bar{x}}_{\varepsilon }-{\bar{c}}_{\varepsilon }\right) ^{n}\right] _{{\textrm{s}}}$. However, from [25, Sec. 2] it follows that we can have $x-c=1$ and $\left[ a_{n\varepsilon }\right] =\left[ {\bar{a}}_{n\varepsilon }\right] =0$ for all $n\in \mathbb {N}$, but

$$\begin{aligned} \left[ \sum _{n=0}^{N_{\varepsilon }}a_{n\varepsilon }(x_{\varepsilon }-c_{\varepsilon })^{n}\right] \ne \left[ \sum _{n=0}^{N_{\varepsilon }}{\bar{a}}_{n\varepsilon }(x_{\varepsilon }-c_{\varepsilon })^{n}\right] . \end{aligned}$$

This means that $(b_{n})_{n}:=\left[ a_{n\varepsilon }(x_{\varepsilon }-c_{\varepsilon })^{n}\right] _{\text {s}}$ and $({\bar{b}}_{n})_{n}:=\left[ {\bar{a}}_{n\varepsilon }(x_{\varepsilon }-c_{\varepsilon })^{n}\right] _{\text {s}}$ yield two different formal HPS (see [25, Theorem 4]) and hence, in general, the operationis not well-defined.

The problem can also be addressed differently: what notion of equality do we have to set on a suitable subring of $\mathbb {R}^{\mathbb {N}\times I}$ so as to have independence on representatives? This notion of equality naturally emerges in proving that the following definition of radius of convergence is well-defined (see Lemma 4). What subring we need to consider arises from the idea to include $\left( \frac{\delta _{\varepsilon }^{(n)}(0)}{n!}\right) _{n,\varepsilon }$in it, where $\delta =\left[ \delta _{\varepsilon }(-)\right]$ is a suitable embedding of Dirac’s delta function (see Example 5.(v)).

### Radius of convergence

The idea to define the radius of convergence corresponding to coefficients $(a_{n\varepsilon })_{n,\varepsilon }\in \mathbb {R}^{\mathbb {N}\times I}$ is that it does not matter if

$$\begin{aligned} \left( \limsup _{n\rightarrow +\infty }\left| a_{n\varepsilon }\right| ^{1/n}\right) ^{-1}\in \mathbb {R}\cup \{+\infty \} \end{aligned}$$

yields a non $\rho$-moderate net (for example for $\varepsilon \in L\subseteq _{0}I$) because this case would intuitively identify a radius of convergence larger than any infinite number in :

#### Definition 3

 

(i) Let ${\overline{\mathbb {R}}}:=\mathbb {R}\cup \{-\infty ,\infty \}$ be the extended real number system with the usual (partially defined) operations but where we define $\infty -\infty =\infty +(-\infty )=-\infty +\infty =-\infty -(-\infty )=0$. We set , where for arbitrary $(x_{\varepsilon })$, $(y_{\varepsilon })\in {\overline{\mathbb {R}}}^{I}$, as usual we define
  $$\begin{aligned} (x_{\varepsilon })\sim _{\rho }(y_{\varepsilon })\quad :\iff \quad \forall q\in \mathbb {N}\,\forall ^{0}\varepsilon :\ \left| x_{\varepsilon }-y_{\varepsilon }\right| \le \rho _{\varepsilon }^{q}. \end{aligned}$$
  Note that, e.g., $(\infty )\sim _{\rho }(\infty )$ because of our definition of $\infty -\infty$. In , we can also consider the standard order relation
  $$\begin{aligned} x\le y\quad :\iff \quad \exists [x_{\varepsilon }]=x,[y_{\varepsilon }]=y\,\forall ^{0}\varepsilon :\ x_{\varepsilon }\le y_{\varepsilon }. \end{aligned}$$
  Note that  is an ordered group but, since we are considering arbitrary nets ${\overline{\mathbb {R}}}^{I}$, the set  is not a ring: e.g. $+\infty \cdot 0$ is still undefined and $+\infty \cdot [z_{\varepsilon }]=[+\infty ]$ for all $(z_{\varepsilon })\in \mathbb {R}_{>0}^{I}$.
(ii) Moreover, we denote by  the quotient ring of *coefficients for HPS*, where
  2.2 $$\begin{aligned} \left( a_{n\varepsilon }\right) _{n,\varepsilon }\in \left( \mathbb {R}^{\mathbb {N}\times I}\right) _{\rho }:\!\iff \exists Q,R\in \mathbb {N}\,\forall ^{0}\varepsilon \,\forall n\in \mathbb {N}:\ \left| a_{n\varepsilon }\right| \le \rho _{\varepsilon }^{-nQ-R} \nonumber \\ \end{aligned}$$
  is the ring of *weakly* $\rho$-*moderate nets*, and
  2.3 $$\begin{aligned} \left( a_{n\varepsilon }\right) _{n,\varepsilon }\simeq _{\rho }\left( {\bar{a}}_{n\varepsilon }\right) _{n,\varepsilon }:\!\iff \forall q,r\in \mathbb {N}\,\forall ^{0}\varepsilon \,\forall n\in \mathbb {N}:\ \left| a_{n\varepsilon }-{\bar{a}}_{n\varepsilon }\right| \le \rho _{\varepsilon }^{nq+r},\nonumber \\ \end{aligned}$$
  in this case, we say that these two nets are *strongly* $\rho$-*equivalent*. Equivalence classes of  are denoted by .
(iii) Finally, if , then we set $\textrm{rad}\left( a_{n}\right) _{\textrm{c}{\varepsilon }}:=r_{\varepsilon }$, and , where
  2.4 $$\begin{aligned} r_{\varepsilon }:=\left( \limsup _{n\rightarrow +\infty }\left| a_{n\varepsilon }\right| ^{1/n}\right) ^{-1}\in \mathbb {R}\cup \{+\infty \}. \end{aligned}$$

In the following lemma, we prove that $\textrm{rad}\left( a_{n}\right) _\textrm{c}$ is well-defined:

#### Lemma 4

Let . Define $r_{\varepsilon }$ as in (2.4) and similarly define ${\bar{r}}_{\varepsilon }$ using ${\bar{a}}_{n\varepsilon }$. Then $(r_{\varepsilon })\sim _{\rho }({\bar{r}}_{\varepsilon })$, and hence $[r_{\varepsilon }]=[{\bar{r}}_{\varepsilon }]$ in .

#### Proof

For all $\varepsilon \in I$ and all $n\in \mathbb {N}_{>0}$, we have $\left| {\bar{a}}_{n\varepsilon }\right| ^{1/n}\le \left( \left| {\bar{a}}_{n\varepsilon } -a_{n\varepsilon }\right| +\left| a_{n\varepsilon }\right| \right) ^{1/n}$. The binomial formula yields $(x+y)\le \left( x^{1/n}+y^{1/n}\right) ^{n}$ for all *x*, $y\in \mathbb {R}_{\ge 0}$, so that $\left| {\bar{a}}_{n\varepsilon }\right| ^{1/n}\le \left| {\bar{a}}_{n\varepsilon } -a_{n\varepsilon }\right| ^{1/n}+\left| a_{n\varepsilon }\right| ^{1/n}$. Setting $r=0$ in (2.3), for all $q\in \mathbb {N}$ and for $\varepsilon$ small we have

$$\begin{aligned} \forall n\in \mathbb {N}:\ \left| a_{n\varepsilon }-{\bar{a}}_{n\varepsilon }\right| \le \rho _{\varepsilon }^{nq}. \end{aligned}$$

Therefore, for the same $\varepsilon$ we get $\left| {\bar{a}}_{n\varepsilon }\right| ^{1/n}\le \rho _{\varepsilon }^{q}+\left| a_{n\varepsilon }\right| ^{1/n}$. Taking the limit superior we obtain $\limsup _{n\rightarrow +\infty }\left| {\bar{a}}_{n\varepsilon }\right| ^{1/n} \le \rho _{\varepsilon }^{q}+\limsup _{n\rightarrow +\infty }\left| a_{n\varepsilon }\right| ^{1/n}$. Inverting the role of $(a_{n\varepsilon })_{n,\varepsilon }$ and $({\bar{a}}_{n\varepsilon })_{n,\varepsilon }$ we finally obtain

$$\begin{aligned} \forall ^{0}\varepsilon :\ -\rho _{\varepsilon }^{q}\le \limsup _{n\rightarrow +\infty }\left| a_{n\varepsilon }\right| ^{1/n} -\limsup _{n\rightarrow +\infty }\left| {\bar{a}}_{n\varepsilon }\right| ^{1/n}\le \rho _{\varepsilon }^{q}, \end{aligned}$$

which proves the claim. $\square$

#### Remark 5

(i) If , then for each fixed $n\in \mathbb {N}$, we have that , i.e. the net $\left( a_{n\varepsilon }\right) _{\varepsilon }$ is $\rho$-moderate. This is the main motivation to consider the exponent “$-R$” in (2.2) (recall that in our notation $0\in \mathbb {N}$): without the term “$-R$”, the only possibility to have  is that $|a_{0}|\le 1$, which is an unnecessary limitation. Similarly, we can motivate why we are considering the quantifier “$\forall n\in \mathbb {N}$” in the same formula (instead of, e.g., “$\exists N\in \mathbb {N}\,\forall n\in \mathbb {N}_{\ge N}$”). The proof of the next Lemma 10 will motivate why in (2.2) we consider the uniform property “$\forall ^{0}\varepsilon \,\forall n\in \mathbb {N}$” and not “$\forall n\in \mathbb {N}\,\forall ^{0}\varepsilon$”.
(ii) Note that  because the notion of equality $\sim _{\rho }$ in the two quotient sets is the same and because if $(x_{\varepsilon })$ is $\rho$-moderate and $(x_{\varepsilon })\sim _{\rho }(y_{\varepsilon })$, then also $(y_{\varepsilon })$ is $\rho$-moderate.
(iii) Condition (2.2) of being weakly $\rho$-moderate represents a constrain on what coefficients $a_{n}$ we can consider in a hyperseries. For example, if $(a_{n})_{n\in \mathbb {N}}$ is a sequence of real numbers satisfying $|a_{n}|\le p(n)$, where $p\in \mathbb {R}[x]$ is a polynomial, then $p(n)\le \rho _{\varepsilon }^{-nQ}$ for all $\varepsilon$ sufficiently small and for all $n\in \mathbb {N}$ if $Q\ge \max \left( 1,\max \left\{ -\frac{\log n}{p(n)\log \rho _{\varepsilon }}\mid n<N_{1}\right\} \right)$, where $\frac{\log n}{P(n)}\le 1$ for all $n\ge N_{1}$ and $-\frac{1}{\log \rho _{\varepsilon }}\le 1$. Hence $\left( a_{n}\right) _{n,\varepsilon }\in \left( \mathbb {R}^{\mathbb {N}\times I}\right) _{\rho }$ is weakly $\rho$-moderate. On the contrary, we cannot have $n^{n}\le \rho _{\varepsilon }^{-nQ-R}=\rho _{\varepsilon }^{-R}\left( \frac{1}{\rho _{\varepsilon }^{Q}}\right) ^{n}$ for all $n\in \mathbb {N}$. Similarly $(n!)_{n\in \mathbb {N}}$ is not weakly $\rho$-moderate and hence our theory does not apply to a “hyperseries” of the form . On the other hand, in Lemma 7.(i) we will show that, as a consequence of considering only weakly moderate coefficients, the radius of convergence of our hyperseries is always strictly positive.
iv Let $a_{n\varepsilon }=\rho _{\varepsilon }^{\frac{n+1}{\varepsilon }}$, so that $\left[ a_{n\varepsilon }\right] _{\text {c}}=0$. The corresponding radius of convergence is $r_{\varepsilon }=\lim _{n\rightarrow +\infty }|a_{n\varepsilon }|^{1/n}=\rho _{\varepsilon }^{1/\varepsilon }$ which is not $\rho$-moderate. In general, if , we can have different behavior on different subpoints, e.g. $r|_{L_{1}}=+\infty$, , $r|_{L_{3}}$ non $\rho$-moderate, etc., where $L_{i}\subseteq _{0}I$. This behavior is studied in Lemma 7 below.
v Let $\mu :=\mathcal {F}^{-1}(\beta )\in \mathcal {S}(\mathbb {R})$ be a Colombeau mollifier defined as the inverse Fourier transform of a smooth, supported in $[-1,1]_{\mathbb {R}}$, even bump function $0\le \beta \le 1$ which identically equals 1 in a neighborhood of 0 (see e.g. [14]). Let $i_{\mathbb {R}}^{b}$ be the embedding of Schwartz distributions into generalized smooth functions (GSF) defined by $\mu$ and by the infinite number  (see e.g. [12]). The Schwartz’s Paley-Wiener theorem implies that $\mu$ is an entire function and we know that if $\textrm{d}\rho ^{-Q}\ge b=[b_{\varepsilon }]\ge \textrm{d}\rho ^{-R}$, for some *Q*, $R\in \mathbb {R}_{>0}$, then the embedding of Dirac delta  is defined by the net $\delta _{\varepsilon }(x)=b_{\varepsilon }\mu (b_{\varepsilon }x)$ (see e.g. [12]). For $n\in \mathbb {N}$, we have $\mu ^{(n)}(0)=\frac{1}{2\pi }\int \beta (x)(ix)^{n}\,\textrm{d} x=0$ if *n* is odd and $\left| \mu ^{(n)}(0)\right| \le \frac{1}{2\pi }\left[ \frac{x^{n+1}}{n+1}\right] _{-1}^{1}\le 1$ if *n* if even. Thereby $\left| \frac{\delta _{\varepsilon }^{(n)}(0)}{n!}\right| =\left| \frac{\mu ^{(n)}(0)}{n!}b_{\varepsilon }^{n+1}\right| \le \frac{1}{n!}\rho _{\varepsilon }^{-nQ-Q}\le \rho _{\varepsilon }^{-nQ-Q}$. This inequality shows that  and motivates our definition of weakly $\rho$-moderate nets. The corresponding radius of convergence is $r_{\varepsilon }^{-1}=\limsup _{n\rightarrow +\infty }\left| \frac{\delta _{\varepsilon }^{(n)}(0)}{n!}\right| ^{1/n} =\limsup _{n\rightarrow +\infty }b_{\varepsilon }^{1+1/n}\left| \frac{\mu ^{(n)}(0)}{n!}\right| ^{1/n}=b_{\varepsilon }\cdot 0=0$, i.e. $\textrm{rad}\left( \frac{\delta ^{(n)}(0)}{n!}\right) _\textrm{c}=+\infty$.
vi et , and assume that for all $\varepsilon$ there exists $r_{\varepsilon }:=\left( \lim _{n\rightarrow +\infty }|a_{n\varepsilon }|^{1/n}\right) ^{-1}$ such that . Then from [21, Theorem 28], for some gauge $\sigma \le \rho$ we have  and . In Corollary 17, we will see the relationship between our definition of radius of convergence and the least upper bound of all the radii where the HPS converges.

In the following lemma, we show that  is a ring:

#### Lemma 6

With pointwise operations,  is a quotient ring.

#### Proof

Actually, the result follows from [13, Theorem 3.6] because the set

$$\begin{aligned} \mathcal {B}:=\left\{ \left( \rho _{\varepsilon }^{-nQ-R}\right) _{n,\varepsilon }\in \mathbb {R}^{\mathbb {N}\times I}\mid Q,\ R\in \mathbb {N}\right\} \end{aligned}$$

is an asymptotic gauge with respect to the order $(n,\varepsilon )\le ({\bar{n}},{\bar{\varepsilon }})$ if and only if $\varepsilon \le {\bar{\varepsilon }}$. However, an independent proof follows the well-known lines of the corresponding proof for the ring , and depends on the following properties of $\mathcal {B}$:

- $$\forall p,q\in \mathcal {B}\,\exists r,s\in \mathcal {B}:\ p+q\le r,\ p\cdot q\le s$$;
- $$\forall p\in \mathcal {B}\,\exists r,s\in \mathcal {B}:\ r^{-1}+s^{-1}\le p^{-1}$$;
- $$\forall p,q,r\in \mathcal {B}\,\exists u,v\in \mathcal {B}:\ u^{-1}\cdot q+v^{-1}\cdot r\le p^{-1}$$,

where $$p=(p_{n\varepsilon })_{n,\varepsilon }\le (q_{n\varepsilon })_{n,\varepsilon }=q$$ means $$\forall ^{0}\varepsilon \,\forall n\in \mathbb {N}:\ p_{n\varepsilon }\le q_{n\varepsilon }$$. $$\square $$

The following lemma represents a useful tool to deal with the radius of convergence. It essentially states that the radius of convergence equals $+\infty$ on some subpoint, or it is moderate on some subpoint or it is greater than any power $\textrm{d}\rho ^{-P}$.

#### Theorem 7

Let  and , then we have

(i) $$r>0$$.
(ii) $$r<+\infty $$ or $$r=_{\textrm{s}}+\infty $$.
(iii) If $r<+\infty$, then the following alternatives hold
  - $$\forall P\in \mathbb {N}:\ r>\textrm{d}\rho ^{-P}$$ or
  - setting
    2.5 $$\begin{aligned}&\left[ r\le \rho ^{-P}\right] :=\left\{ \varepsilon \mid r_{\varepsilon } \le \rho _{\varepsilon }^{-P}\right\} =:L_{P}\nonumber \\&P_{\text {m}}:=\min \left\{ P\in \mathbb {N}\mid \left[ r\le \rho ^{-P}\right] \subseteq _{0}I\right\} \end{aligned}$$
    we have
    1. $$I=\bigcup _{P\in \mathbb {N}}\left[ r\le \rho ^{-P}\right] $$;
    2. $$\forall P\ge P_{\text {m}}:\ \left[ r\le \rho ^{-P}\right] \subseteq _{0}I,\ r\le _{L_{P}}\textrm{d}\rho ^{-P}$$;
    3. $$\forall P<P_{\text {m}}:\ \textrm{d}\rho ^{-P}\le r$$;
    4. If $P_{\text {m}}=0$ and $L_{0}^{c}\subseteq _{0}I$, then $1\le _{L_{0}^{c}}r$; if $L_{0}^{c}\not \subseteq _{0}I$, then $r\le 1$.
(iv) Assume that for all $L\subseteq _{0}I$, the following implication holds
  2.6 $$\begin{aligned} \left( \exists Q\in \mathbb {N}:\ r\le _{L}\textrm{d}\rho ^{-Q}\right) \text { or }\left( \forall Q\in \mathbb {N}:\ r>_{L}\textrm{d}\rho ^{-Q}\right) \ \Rightarrow \ \forall ^{0}\varepsilon \in L:\ \mathcal {P}\left\{ r_{\varepsilon }\right\} .\nonumber \\ \end{aligned}$$
  Then $\forall ^{0}\varepsilon :\ \mathcal {P}\left\{ r_{\varepsilon }\right\}$, i.e. the property $\mathcal {P}\left\{ r_{\varepsilon }\right\}$ holds for all sufficiently small $\varepsilon$.
(v) If  and $q<r$, then $\exists s$.

#### Proof

(i): Assume that $\left| a_{n\varepsilon }\right| \le \rho _{\varepsilon }^{-nQ-R}$ for all $\varepsilon \le \varepsilon _{0}$ and for all $n\in \mathbb {N}$. Then $\limsup _{n\rightarrow +\infty }\left| a_{n\varepsilon }\right| ^{1/n}\le \lim _{n\rightarrow +\infty } \rho _{\varepsilon }^{-Q-\frac{R}{n}}=\rho _{\varepsilon }^{-Q}$, i.e. $r_{\varepsilon }\ge \rho _{\varepsilon }^{Q}$.

(ii): Set $L:=\left\{ \varepsilon \mid r_{\varepsilon }=+\infty \right\}$. If $L\subseteq _{0}I$, then $r=_{L}+\infty$. Otherwise $(0,\varepsilon _{0}]\cap L=\emptyset$ for some $\varepsilon _{0}$, i.e. $r_{\varepsilon }<+\infty$ for all $\varepsilon \le \varepsilon _{0}$.

(iii): Since we assume that $r<+\infty$, without loss of generality we can take $r_{\varepsilon }<+\infty$ for all $\varepsilon$. We also assume that (a) is false, i.e. $r\le _{M}\textrm{d}\rho ^{-{\bar{P}}}$ for some ${\bar{P}}\in \mathbb {N}$ and some $M\subseteq _{0}I$. We first prove (b.1): take $\varepsilon \in \bigcap _{P\in \mathbb {N}}\left[ r>\rho ^{-P}\right]$, then $r_{\varepsilon }>\rho _{\varepsilon }^{-P}$ for all $P\in \mathbb {N}$, so that $r_{\varepsilon }=+\infty$ for $P\rightarrow +\infty$, and this is not possible. We also note that $\left[ r\le \rho ^{-P}\right] \subseteq \left[ r\le \rho ^{-Q}\right]$ for all $Q\ge P$. From $M\subseteq _{0}I$ and $r\le _{M}\textrm{d}\rho ^{-{\bar{P}}}$, we have $(0,\varepsilon _{0}]\cap M\subseteq \left[ r\le \rho ^{-({\bar{P}}+1)}\right] \subseteq _{0}I$, and hence definition (2.5) yields $P_{\text {m}}\in \mathbb {N}$ and also proves (b.2). For all $P\in \mathbb {N}_{<P_{\text {m}}}$, we hence have $\left[ r\le \rho ^{-P}\right] \not \subseteq _{0}I$, i.e. $(0,\varepsilon _{P}]\subseteq \left[ r>\rho ^{-P}\right]$ for some $\varepsilon _{P}$. This implies $\textrm{d}\rho ^{-P}\le r$ and proves (b.3). Finally, if $P_{\text {m}}=0$ and $L_{P_{\text {m}}}^{c}=L_{0}^{c}\subseteq _{0}I$, then $1\le _{L_{0}^{c}}r$ because $L_{0}^{c}=\left[ r>1\right]$. If $L_{0}^{c}\not \subseteq _{0}I$, then $(0,\varepsilon _{0}]\subseteq L_{0}$ for some $\varepsilon _{0}$, i.e. $r\le 1$.

(iv): By contradiction, assume that $\lnot \mathcal {P}\left\{ r_{\varepsilon }\right\}$ for all $\varepsilon \in L$ and for some $L\subseteq _{0}I$. As usual, we assume that all the results we proved for  can also be similarly proved for the restriction . From (ii) for , we have $r<_{L}+\infty$ or $r=_{K}+\infty$ for some $K\subseteq _{0}L$. The second case implies $r>_{L}\textrm{d}\rho ^{-Q}$ for all $Q\in \mathbb {N}$. Since $K\subseteq _{0}I$, we can apply the second alternative in the implication (2.6) to get $\forall ^{0}\varepsilon \in K:\ \mathcal {P}\left\{ r_{\varepsilon }\right\}$, which gives a contradiction because $K\subseteq L$. We can hence consider the first case $r<_{L}+\infty$ and apply the subcase (a), i.e. $r>_{L}\textrm{d}\rho ^{-P}$ for all $P\in \mathbb {N}$, and we hence proceed as above applying the second alternative of the implication (2.6). In the remaining subcase, we can use (b.2) (with *L* instead of *I*). This yields $L_{P_{\text {m}}}\subseteq _{0}L$ and $r\le _{L_{P_{\text {m}}}}\textrm{d}\rho ^{-P_{\text {m}}}$. Since $L_{P_{\text {m}}}\subseteq _{0}I$, we can apply the first alternative in the implication (2.6) to get once again a contradiction.

(v): Assume that $r>q$ and take . $\square$

Explicitly note the meaning of Lemma 7(iv): on an arbitrary subpoint $r|_{L}$ of the radius of convergence $r=\textrm{rad}\left( a_{n}\right) _\textrm{c}$, we have to consider only two cases: either $r|_{L}$ is $\rho$-moderate or it is greater than any power $\textrm{d}\rho ^{-Q}$ (the latter case including also the case $r|_{L}=+\infty$); if in both cases we are able to prove the property $\mathcal {P}\left\{ r_{\varepsilon }\right\}$ for $\varepsilon \in L$ sufficiently small, then this property holds for *all* $\varepsilon$ sufficiently small.

### Set of convergence

Even if the radius of convergence of the exponential hyperseries is $\textrm{rad}\left( \frac{1}{n!}\right) _\textrm{c}=+\infty$, we have that  implies $|x|\le \log \left( \textrm{d}\rho ^{-R}\right)$ for some $R\in \mathbb {N}$: in other words, the constraint to get a $\rho$-moderate number implies that even if  converges at *x*, the exponential HPS does *not* converge in the interval .

Moreover, in all our examples, if the HPS  converges, then it converges exactly to . The following definition of *set of convergence* closely recalls the definition of GSF:

#### Definition 8

Let  and . The set of convergenceis the set of all  satisfying

(i) $$\left| x-c\right| <\textrm{rad}\left( a_{n}\right) _\textrm{c}$$,

and such that there exist representatives $[x_{\varepsilon }]=x$, $\left[ a_{n\varepsilon }\right] _{{\textrm{c}}}=\left( a_{n}\right) _{{\textrm{c}}}$ and $[c_{\varepsilon }]=c$ satisfying the following conditions:

(ii), i.e. we have a formal HPS;
(iii);
(iv) For all representatives $[{\bar{x}}_{\varepsilon }]=x$ and all $k\in \mathbb {N}_{>0}$, the *k*-th derivative net is $\rho$-moderate:
  $$\begin{aligned} \left( \frac{\textrm{d} ^{k}}{\textrm{d}{x}^{k}}\left( \sum _{n=0}^{+\infty }a_{n\varepsilon }(x-c_{\varepsilon })^{n}\right) _{x={\bar{x}}_{\varepsilon }}\right) \in \mathbb {R}_{\rho }. \end{aligned}$$

Note that condition (ii) is necessary because in (iii) we use a HPS; on the other hand, conditions (iii) and (iv) state that the functionis a GSF defined by the net of smooth functions $\left( \sum _{n=0}^{+\infty }a_{n\varepsilon }(x_{\varepsilon }-c_{\varepsilon })^{n}\right)$. As for GSF, see [12, Theorem 16], condition (iv) will be useful to prove that we have independence from representatives of *x* in all the derivatives. In Corollary 25, we will see that under very general assumptions and if $\sigma \le \rho ^{*}$, condition (iv) can be omitted.

In Sect. 14 we will show that  (the set of convergence of the exponential HPS at the origin), but $\textrm{d}\rho ^{-1}\notin \text {conv}\left( \left( \frac{1}{n!}\right) _{n}^{{\textrm{c}}},0\right)$. We immediately note that $x\in \text {conv}\left( \left( a_{n}\right) _{n}^{\text {c}},c\right)$ if and only if $x-c\in \text {conv}\left( \left( a_{n}\right) _{n}^{\text {c}},0\right)$, and because of this property without loss of generality we will frequently assume $c=0$.

We also note that condition (iii) states that the hyperseries  converges, and it does exactly to the generalized number $\left[ \sum _{n=0}^{+\infty }a_{n\varepsilon }x_{\varepsilon }^{n}\right]$. It is hence natural to wonder whether it is possible that it converges to some different quantity. This is the problem of the relation between hyperlimit and $\varepsilon$-wise limit:which has been already addressed in [25, Theorems 12, 13]. Intuitively speaking, if the gauge $(\sigma _{\varepsilon })$ is not sufficiently small, and hence the infinite nets $(\sigma _{\varepsilon }^{-N})$ are not sufficiently large, it can happen that ${ni}{(N)}_{\varepsilon }\rightarrow +\infty$ as $\varepsilon \rightarrow 0$ only very slowly, whereas the $\varepsilon$-wise limit could require $N\rightarrow +\infty$ at a greater speed to converge. This can be stated more precisely in the following way: Let  be a formal HPS and assume that $\sum _{n=0}^{+\infty }a_{n\varepsilon }x_{\varepsilon }^{n}<+\infty$ for $\varepsilon$ small. Then, for all $q\in \mathbb {N}$ and for all $\varepsilon$ small, we can find $N_{\varepsilon }^{q}\in \mathbb {N}$ such that

$$\begin{aligned} \left| \sum _{n=0}^{N_{\varepsilon }^{q}}a_{n\varepsilon }x_{\varepsilon }^{n}-\sum _{n=0}^{+\infty } a_{n\varepsilon }x_{\varepsilon }^{n}\right| \le \rho _{\varepsilon }^{q}\quad \forall n\in \mathbb {N}_{\ge N_{\varepsilon }^{q}}. \end{aligned}$$

However, only if $\left( \sum _{n=0}^{+\infty }a_{n\varepsilon }x_{\varepsilon }^{n}\right) \in \mathbb {R}_{\rho }$ and $(N_{\varepsilon }^{q})\in \mathbb {R}_{\sigma }$, i.e. , then this also implies .

As expected, for HPS the set of convergence is never a singleton:

#### Theorem 9

Let  and . Then2.7

#### Proof

From Theorem 7(i), we have $r:=\textrm{rad}\left( a_{n}\right) _\textrm{c}\ge \textrm{d}\rho ^{q_{1}}$ for some $q_{1}\in \mathbb {N}$. We also have $|a_{n\varepsilon }|\le \rho _{\varepsilon }^{-nQ-R}$ from (2.2). Assume that $|x-c|<\textrm{d}\rho ^{q}$: we want to find $q\in \mathbb {N}_{\ge q_{1}}$ so that . To prove property Definition 8(ii), for $N_{\varepsilon }$, $M_{\varepsilon }\in \mathbb {N}$ and for $\varepsilon$ small, we estimate

$$\begin{aligned} \left| \sum _{n=N_{\varepsilon }}^{M_{\varepsilon }}a_{n\varepsilon }(x_{\varepsilon }-c_{\varepsilon })^{n}\right| \le \sum _{n=N_{\varepsilon }}^{M_{\varepsilon }}\rho _{\varepsilon }^{-nQ-R}\rho _{\varepsilon }^{nq}=\rho _{\varepsilon }^{-R} \sum _{n=N_{\varepsilon }}^{M_{\varepsilon }}\rho _{\varepsilon }^{-nQ+nq}. \end{aligned}$$

Therefore, taking $q=\max (1+Q,q_{1})$, we get

$$\begin{aligned} \left| \sum _{n=N_{\varepsilon }}^{M_{\varepsilon }}a_{n\varepsilon }(x_{\varepsilon }-c_{\varepsilon })^{n}\right| \le \rho _{\varepsilon }^{-R}\sum _{n=N_{\varepsilon }}^{M_{\varepsilon }}\rho _{\varepsilon }^{n} \le \frac{\rho _{\varepsilon }^{-R}}{1-\rho _{\varepsilon }}, \end{aligned}$$

and this proves Definition 8(ii). Similarly, we have

$$\begin{aligned} \left| \sum _{n=0}^{M_{\varepsilon }}a_{n\varepsilon }(x_{\varepsilon }-c_{\varepsilon })^{n} -\sum _{n=0}^{+\infty }a_{n\varepsilon }(x_{\varepsilon }-c_{\varepsilon })^{n}\right|&\le \sum _{n=M_{\varepsilon }+1}^{+\infty }\rho _{\varepsilon }^{n}\le \frac{\rho _{\varepsilon }^{M_{\varepsilon }+1}}{1-\rho _{\varepsilon }}. \end{aligned}$$

Since , this proves Definition 8(iii). Finally, for all $k\in \mathbb {N}_{>0}$ and all representatives $[{\bar{x}}_{\varepsilon }]=x$, we have

2.8 $$\begin{aligned} \frac{\textrm{d} ^{k}}{\textrm{d}{x}^{k}}\left( \sum _{n=0}^{+\infty }a_{n\varepsilon }(x-c_{\varepsilon })^{n}\right) _{x={\bar{x}}_{\varepsilon }}&=\sum _{n=k}^{+\infty }a_{n\varepsilon }({\bar{x}}_{\varepsilon }-c_{\varepsilon })^{n-k}\prod _{j=0}^{k-1}(n-j) \end{aligned}$$

2.9 $$\begin{aligned}&=k!\sum _{n=k}^{+\infty }a_{n\varepsilon }({\bar{x}}_{\varepsilon }-c_{\varepsilon })^{n-k}{n \atopwithdelims ()k}, \end{aligned}$$

and hence

$$\begin{aligned} \left| \frac{\textrm{d} ^{k}}{\textrm{d}{x}^{k}}\left( \sum _{n=0}^{+\infty }a_{n\varepsilon } ({\bar{x}}_{\varepsilon }-c_{\varepsilon })^{n}\right) \right|&\le \sum _{n=k}^{+\infty } \rho _{\varepsilon }^{-nQ-R}\rho _{\varepsilon }^{(n-k)q}\prod _{j=0}^{k-1}(n-j)\\&=\rho _{\varepsilon }^{-R-kQ}\sum _{n=k}^{+\infty }\rho _{\varepsilon }^{(q-Q)(n-k)}\prod _{j=0}^{k-1}(n-j)\\&=\rho _{\varepsilon }^{-R-kQ}\frac{k!}{(1-\rho _{\varepsilon }^{q-Q})^{k+1}}\in \mathbb {R}_{\rho }. \end{aligned}$$

In the last step we used $q\ge Q+1$ and the binomial series $\sum _{n=k}^{+\infty }y^{n-k}\prod _{j=0}^{k-1}(n-j)=k!\sum _{n=k}^{+\infty }{n \atopwithdelims ()k}y^{n-k}=\frac{k!}{(1-y)^{k+1}}$ for $|y|<1$. $\square$

We can now prove independence from representatives both in Definition 8 and in Definition 1:

#### Lemma 10

Let , $x=[x_{\varepsilon }]=[{\bar{x}}_{\varepsilon }]$, . Assume that . Then

(i) The nets $\left( a_{n\varepsilon }\right) _{n,\varepsilon }$, $(x_{\varepsilon })$ and $(c_{\varepsilon })$ also satisfy all the conditions of Definition 8 of set of convergence.
(ii) $\left[ a_{n\varepsilon }\cdot \left( x_{\varepsilon }-c_{\varepsilon }\right) ^{n}\right] _{{\textrm{s}}}=\left[ {\bar{a}}_{n\varepsilon }\cdot \left( {\bar{x}}_{\varepsilon }-{\bar{c}}_{\varepsilon }\right) ^{n}\right] _{{\textrm{s}}}$, where the equality is in .

#### Proof

(i): Since we have similar steps for several claims, let $N_{\varepsilon }\in \mathbb {N}$ and $M_{\varepsilon }\in \mathbb {N}\cup \{+\infty \}$, so that a term of the form $\sum _{n=N_{\varepsilon }}^{M_{\varepsilon }}b_{n\varepsilon }$ represents both the ordinary series $\sum _{n=0}^{+\infty }b_{n\varepsilon }$ or the finite sum $\sum _{n=N_{\varepsilon }}^{M_{\varepsilon }}b_{n\varepsilon }$. From Definition 8 of set of convergence, we get the existence of representatives , $\left[ {\hat{a}}_{n\varepsilon }\right] _{{\textrm{c}}}=\left( a_{n}\right) _{n}^{{\textrm{c}}}$ and $[{\hat{c}}_{\varepsilon }]=c$ satisfying Definition 8. Set ${\hat{y}}_{\varepsilon }:={\hat{x}}_{\varepsilon }-{\hat{c}}_{\varepsilon }$, ${\hat{y}}:=[{\hat{y}}_{\varepsilon }]$. Let $r:=[r_{\varepsilon }]:=\textrm{rad}\left( a_{n}\right) _\textrm{c}$ be the radius of convergence. From Lemma 7(v), take  satisfying $|{\hat{y}}|<s\le r$ and a representative $[s_{\varepsilon }]=s$ such that $|{\hat{y}}_{\varepsilon }|<s_{\varepsilon }\le r_{\varepsilon }$ for all $\varepsilon$ small. Set $z_{n\varepsilon }:=a_{n\varepsilon }-{\hat{a}}_{n\varepsilon }$ and ${\hat{z}}_{\varepsilon }:=y_{\varepsilon }-{\hat{y}}_{\varepsilon }$. For all $k\in \mathbb {N}$, we have

2.10 $$\begin{aligned} \sum _{n=N_{\varepsilon }}^{M_{\varepsilon }}a_{n\varepsilon }y_{\varepsilon }^{n-k}{n \atopwithdelims ()k}&=\sum _{n=N_{\varepsilon }}^{M_{\varepsilon }}\left( {\hat{a}}_{n\varepsilon }+z_{n\varepsilon }\right) \left( {\hat{y}}_{\varepsilon }+{\hat{z}}_{\varepsilon }\right) ^{n-k}{n \atopwithdelims ()k}\nonumber \\&=\sum _{n=N_{\varepsilon }}^{M_{\varepsilon }}{\hat{a}}_{n\varepsilon }\left( {\hat{y}}_{\varepsilon } +{\hat{z}}_{\varepsilon }\right) ^{n-k}{n \atopwithdelims ()k}+\sum _{n=N_{\varepsilon }}^{M_{\varepsilon }}z_{n\varepsilon }\left( {\hat{y}}_{\varepsilon } +{\hat{z}}_{\varepsilon }\right) ^{n-k}{n \atopwithdelims ()k}. \end{aligned}$$

Since ${\hat{z}}=0$, we also have $|{\hat{y}}_{\varepsilon }|+|{\hat{z}}_{\varepsilon }|<s_{\varepsilon }\le r_{\varepsilon }$ for all $\varepsilon$ small. For the same $\varepsilon$, assume that $|z_{n\varepsilon }|\le \rho _{\varepsilon }^{np+q}$ for fixed arbitrary *p*, $q\in \mathbb {N}$. We first consider the second summand in (2.10):

$$\begin{aligned} \left| \sum _{n=N_{\varepsilon }}^{M_{\varepsilon }}z_{n\varepsilon }\left( {\hat{y}}_{\varepsilon } +{\hat{z}}_{\varepsilon }\right) ^{n-k}{n \atopwithdelims ()k}\right| \le \sum _{n=N_{\varepsilon }}^{M_{\varepsilon }} \rho _{\varepsilon }^{np+q}s_{\varepsilon }^{n-k}{n \atopwithdelims ()k}=\rho _{\varepsilon }^{q+kp} \sum _{n=N_{\varepsilon }}^{M_{\varepsilon }}\left( \rho _{\varepsilon }^{p}s_{\varepsilon }\right) ^{n-k}{n \atopwithdelims ()k}\\ \le \rho _{\varepsilon }^{q+kp}\sum _{n=k}^{+\infty }\left( \rho _{\varepsilon }^{p}s_{\varepsilon }\right) ^{n-k}{n \atopwithdelims ()k}-\rho _{\varepsilon }^{q+kp}\sum _{n=k}^{N_{\varepsilon }-1} \left( \rho _{\varepsilon }^{p}s_{\varepsilon }\right) ^{n-k}{n \atopwithdelims ()k}\\ \le 2\rho _{\varepsilon }^{q+kp}\sum _{n=k}^{+\infty }\left( \rho _{\varepsilon }^{p}s_{\varepsilon }\right) ^{n-k}{n \atopwithdelims ()k}. \end{aligned}$$

Since , we can take $p\in \mathbb {N}$ sufficiently large so that $\rho _{\varepsilon }^{p}s_{\varepsilon }<1$. This implies

$$\begin{aligned} \left| \sum _{n=N_{\varepsilon }}^{M_{\varepsilon }}z_{n\varepsilon }\left( {\hat{y}}_{\varepsilon }+{\hat{z}}_{\varepsilon }\right) ^{n-k}{n \atopwithdelims ()k}\right| \le \frac{2\rho _{\varepsilon }^{q+kp}}{\left( 1-\rho _{\varepsilon }^{p}s_{\varepsilon }\right) ^{k+1}}. \end{aligned}$$

Thereby, for $q\rightarrow +\infty$, this summand defines a negligible net. For the first summand of (2.10), we can use the mean value theorem to get

2.11 $$\begin{aligned}  &   \left| \sum _{n=N_{\varepsilon }}^{M_{\varepsilon }}{\hat{a}}_{n\varepsilon }\left( {\hat{y}}_{\varepsilon } +{\hat{z}}_{\varepsilon }\right) ^{n-k}{n \atopwithdelims ()k}-\sum _{n=N_{\varepsilon }}^{M_{\varepsilon }} {\hat{a}}_{n\varepsilon }{\hat{y}}_{\varepsilon }^{n-k}{n \atopwithdelims ()k}\right| \nonumber \\  &   \quad \le \left| \sum _{n=N_{\varepsilon }}^{M_{\varepsilon }}{\hat{a}}_{n\varepsilon }(n-k)\xi _{\varepsilon }^{n-k-1}{n \atopwithdelims ()k}{\hat{z}}_{\varepsilon }\right| =\left| {\hat{z}}_{\varepsilon }\right| \left| \sum _{n=N_{\varepsilon }}^{M_{\varepsilon }}{\hat{a}}_{n\varepsilon }(n-k)\xi _{\varepsilon }^{n-k-1}{n \atopwithdelims ()k}\right| \nonumber \\ \end{aligned}$$

for some $\xi _{\varepsilon }\in [{\hat{y}}_{\varepsilon },{\hat{y}}_{\varepsilon }+{\hat{z}}_{\varepsilon }]\cup [{\hat{y}}_{\varepsilon }+{\hat{z}}_{\varepsilon },{\hat{y}}_{\varepsilon }]$. Thereby, the right hand side of (2.11) is negligible because of Definition 8(iv).

We can hence state that for all $k\in \mathbb {N}$

2.12 $$\begin{aligned} \left( \sum _{n=N_{\varepsilon }}^{M_{\varepsilon }}a_{n\varepsilon }\left( y_{\varepsilon }+z_{\varepsilon }\right) ^{n-k}{n \atopwithdelims ()k}\right) \sim _{\rho }\left( \sum _{n=N_{\varepsilon }}^{M_{\varepsilon }}{\hat{a}}_{n\varepsilon }{\hat{y}}_{\varepsilon }^{n-k}{n \atopwithdelims ()k}\right) . \nonumber \\ \end{aligned}$$

In the case $M_{\varepsilon }<+\infty$ for all $\varepsilon$ and $k=0$, this proves that  because $({\hat{a}}_{n\varepsilon })_{n,\varepsilon }$ and $({\hat{y}}_{\varepsilon })$ satisfy Definition 8(ii). In the case $M_{\varepsilon }=+\infty$ and $N_{\varepsilon }=0=k$, it proves the moderateness of $\left( \sum _{n=0}^{+\infty }a_{n\varepsilon }y_{\varepsilon }^{n}\right)$ too, i.e. the implicit moderateness requirement of Definition 8(iii). Finally, for $k>0$, from property (2.12) we have that Definition 8(iv) holds for $(a_{n\varepsilon })$ and $(y_{\varepsilon })$ because of (2.8). We can apply (2.12) with $k=0$ to $\left[ {\bar{a}}_{n\varepsilon }\right] _{{\textrm{c}}}=\left( a_{n}\right) _{{\textrm{c}}}$, $[{\bar{x}}_{\varepsilon }]=x$, $[{\bar{c}}_{\varepsilon }]=c$, ${\bar{y}}_{\varepsilon }:={\bar{x}}_{\varepsilon }-{\bar{c}}_{\varepsilon }$ and with $M_{\varepsilon }<+\infty$, to get

$$\begin{aligned} \left( \sum _{n=N_{\varepsilon }}^{M_{\varepsilon }}a_{n\varepsilon }y_{\varepsilon }^{n}\right) \sim _{\rho }\left( \sum _{n=N_{\varepsilon }}^{M_{\varepsilon }}{\hat{a}}_{n\varepsilon }{\hat{y}}_{\varepsilon }^{n}\right) \sim _{\rho }\left( \sum _{n=N_{\varepsilon }}^{M_{\varepsilon }}{\bar{a}}_{n\varepsilon }{\bar{y}}_{\varepsilon }^{n}\right) . \end{aligned}$$

This proves claim (ii) and hence also Definition 8(iii) because  converges to  from (2.12). $\square$

### Examples

We start studying geometric hyperseries, which in general are convergent HPS if $\sigma \le \rho ^{*}$:

#### Example 11

(Geometric hyperseries) Assume that . We have:2.13This shows that  for all gauges $\rho$, $\sigma$. Hence by Definition 1, , i.e. the geometric series is a formal hyper-series. Since coefficients $a_{n\varepsilon }=1$, we have  (see Definition 3(i)). Now, by Definition 3(iii), $\textrm{rad}\left( 1\right) _\textrm{c}=1$. From Definition 8(i), we have . Now, take $x=[x_{\varepsilon }]\in (-1,1)$, with $-1<x_{\varepsilon }<1$ for all $\varepsilon$. From [25, Example 8], if $\sigma \le \rho ^{*}$ (i.e. if $\exists Q\in \mathbb {R}_{>0}\,\forall ^{0}\varepsilon :\ \sigma _{\varepsilon }\le \rho _{\varepsilon }^{Q}$), we have Definition 8(iii). Finally, if $[{\bar{x}}_{\varepsilon }]=x$ is another representative and $k\in \mathbb {N}_{>0}$, then $-1<{\bar{x}}_{\varepsilon }<1$ for $\varepsilon$ small, and from (2.8) we get $\sum _{n=k}^{+\infty }k!{n \atopwithdelims ()k}{\bar{x}}_{\varepsilon }^{n-k}=\frac{k!}{(1-{\bar{x}}_{\varepsilon })^{k+1}}\in \mathbb {R}_{\rho }$ because $1-x>0$ is invertible.

Note explicitly that $\sigma \le \rho ^{*}$ is a sufficient condition ensuring the convergence of *any* geometric hyperseries with $|x|<1$. However, we already used (see e.g. Theorem 9) the convergence of the geometric hyperseries  for all gauges $\rho$, $\sigma$. More generally, exactly as proved in [25, Example 8], it is easy to see that  if $\sigma _{\varepsilon }\le \left( \frac{\log x_{\varepsilon }}{\log \rho _{\varepsilon }}\right) ^{Q}$ for $\varepsilon$ small and some $Q\in \mathbb {R}_{>0}$.

#### Example 12

(A smooth function with a flat point) Consider the GSF corresponding to the ordinary smooth function

$$\begin{aligned} f(x):={\left\{ \begin{array}{ll} e^{-1/x} &  \text {if }x\in \mathbb {R}_{>0}\\ 0 &  \text {otherwise} \end{array}\right. }. \end{aligned}$$

It is not hard to prove that $|f(x)|\le |x|^{q}$ for all $x\approx 0$ and all $q\in \mathbb {N}$. Thereby, $f(x)=0$ for all *x* such that $|x|\le \textrm{d}\rho ^{r}$ for some $r\in \mathbb {R}_{>0}$. Therefore, we trivially have  only for all *x* in this infinitesimal neighborhood of 0. On the other hand, . Moreover, $\textrm{rad}\left( \frac{f^{(n)}(c)}{n!}\right) _\textrm{c}=+\infty$ and  for all  such that $|c|\gg 0$, i.e. satisfying $|c|\ge r$ for some $r\in \mathbb {R}_{>0}$, but  only for all  such that $|x|\gg 0$, which is a strict subset of . The GSF *f* is therefore a candidate to be a GRAF, but not an entire GRAF.

#### Example 13

(A nowhere analytic smooth function)

A classical example of an infinitely differentiable function which is not analytic at any point is $F(x)=\sum _{k\in 2^{\mathbb {N}}}e^{-\sqrt{k}}\cos (kx)$, where $2^{\mathbb {N}}:=\left\{ 2^{n}\mid n\in \mathbb {N}\right\}$. Since for all $x=\pi \frac{p}{q}$, with $p\in \mathbb {N}$ and $q\in 2^{\mathbb {N}}$ and for all $n\in 2^{\mathbb {N}}$, $n\ge 4$, $n>q$, we have $F^{(n)}(x)\ge e^{-2n}(4n^{2})^{n}+O(q^{n})$ as $n\rightarrow +\infty$, we have that $\left( \frac{F^{(n)}(x)}{n!}\right) _{n,\varepsilon }\notin \left( \mathbb {R}^{\mathbb {N}\times I}\right) _{\rho }$, i.e. they are *not* coefficients for a HPS. Note that this also proves that not even all smooth functions can be embedded as GRAF.

#### Example 14

(Exponential) We clearly have  and $\textrm{rad}\left( \frac{1}{n!}\right) _\textrm{c}=+\infty$, i.e. we have coefficients for an HPS with infinite radius of convergence. SetFor all $x=[x_{\varepsilon }]\in C$ and all $N_{\varepsilon }$, $M_{\varepsilon }\in \mathbb {N}$, we have $\left| \sum _{n=N_{\varepsilon }}^{M_{\varepsilon }}\frac{x_{\varepsilon }^{n}}{n!}\right| \le e^{|x_{\varepsilon }|}\le \rho _{\varepsilon }^{-K}$ for $\varepsilon$ small, and this shows that , i.e. for all $x\in C$, we have a formal HPS. We finally want to prove that  if $\sigma \le \rho ^{*}$. The inclusion $\supseteq$ follows directly from Definition 8(iii). If $x=[x_{\varepsilon }]\in C$, then condition Definition 8(iv) holds because the *k*-th derivative HPS $\left( k!\sum _{n=k}^{+\infty }{n \atopwithdelims ()k}\cdot \frac{x_{\varepsilon }^{n-k}}{n!}\right) =\left( e^{x_{\varepsilon }}\right) \in \mathbb {R}_{\rho }$. To prove Definition 8(iii), assume that $|x_{\varepsilon }|<-K\log \rho _{\varepsilon }=:M_{\varepsilon }$ for all $\varepsilon$ and set . Take  such that $\frac{M}{N+1}<\frac{1}{2}$, so that, exactly as in [25, Example 8], we can prove that $\frac{M^{n+1}}{(n+1)!}<\frac{1}{2^{n+1}}$ and hence $\left| \sum _{n=N_{\varepsilon }+1}^{+\infty }\frac{x_{\varepsilon }^{n}}{n!}\right| \le \sum _{n\ge N_{\varepsilon }}\frac{1}{2^{n}}\rightarrow 0$ as $N\rightarrow +\infty$, , if $\sigma \le \rho ^{*}$. Similarly, we can consider trigonometric functions whose set of convergence is the whole of .

#### Example 15

(*Dirac delta*) In Remark 5(v), we already proved that  and $\textrm{rad}\left( \frac{\delta ^{(n)}(0)}{n!}\right) _\textrm{c}=+\infty$. For all  and all $N_{\varepsilon }$, $M_{\varepsilon }\in \mathbb {N},$ we have $\left| \sum _{n=N_{\varepsilon }}^{M_{\varepsilon }}\frac{\delta _{\varepsilon }^{(n)}(0)}{n!}x_{\varepsilon }^{n}\right| \le b_{\varepsilon }\cdot \sum _{n=0}^{+\infty }\frac{|\mu ^{(n)}(0)|}{n!}|b_{\varepsilon }x_{\varepsilon }|^{n}$. But $|\mu ^{(n)}(0)|=i^{n}\mu ^{(n)}(0)$ because $\mu ^{(n)}(0)=0$ if *n* is odd, so that $\left| \sum _{n=N_{\varepsilon }}^{M_{\varepsilon }}\frac{\delta _{\varepsilon }^{(n)}(0)}{n!}x_{\varepsilon }^{n}\right| \le b_{\varepsilon }\cdot \sum _{n=0}^{+\infty }\frac{\mu ^{(n)}(0)}{n!}|ib_{\varepsilon }x_{\varepsilon }|^{n} =b_{\varepsilon }\mu (i|b_{\varepsilon }x_{\varepsilon }|)\in \mathbb {R}_{\rho }$, and this proves that , i.e. we always have a formal HPS. Condition Definition 8(iv) follows because derivatives are always moderate. It remains to prove Definition 8(iii) for all  to show that :

$$\begin{aligned} \sum _{n=0}^{N}\frac{\delta ^{(n)}(0)}{n!}x^{n}=\left[ b_{\varepsilon }\sum _{n=0}^{N_{\varepsilon }} \frac{\mu ^{(n)}(0)}{n!}b_{\varepsilon }^{n}x_{\varepsilon }^{n}\right] =\delta (x)-b\left[ \mu ^{(N_{\varepsilon }+1)} ({\bar{x}}_{\varepsilon })\frac{x_{\varepsilon }^{N_{\varepsilon }+1}}{(N_{\varepsilon }+1)!}\right] \end{aligned}$$

where the existence of ${\bar{x}}_{\varepsilon }\in [0,x_{\varepsilon }]\cup [x_{\varepsilon },0]$ is derived from Taylor’s formula. Since $|\mu ^{(k)}(y)|\le \frac{1}{2\pi }\int \beta (x)|x|^{k}\,\textrm{d} x=:C\in \mathbb {R}_{>0}$ for all $k\in \mathbb {N}$ and all $y\in \mathbb {R}$, we obtain

$$\begin{aligned} \left| \sum _{n=0}^{N}\frac{\delta ^{(n)}(0)}{n!}x^{n}-\delta (x)\right| \le bC\left[ \frac{|x_{\varepsilon }|^{N_{\varepsilon }+1}}{(N_{\varepsilon }+1)!}\right] . \end{aligned}$$

Using Stirling’s approximation, we have $\frac{|x_{\varepsilon }|^{N_{\varepsilon }+1}}{(N_{\varepsilon }+1)!}\le 2\left( \frac{|x_{\varepsilon }|e}{N_{\varepsilon }}\right) ^{N}\le \rho _{\varepsilon }^{N_{\varepsilon }}$ for all  such that $N>|x|e\textrm{d}\rho ^{-1}$, which is always possible if $\sigma \le \rho ^{*}$. Since , this proves the claim.

A different way to include a large class of examples is to use the characterization Theorem 37 by factorial growth of derivatives of GRAF.

When we say that a HPS  is *convergent*, we already assume that its coefficients are correctly chosen and that the point *x* is in the set of convergence, as stated in the following

#### Definition 16

We say that  is a *convergent* HPS if

(i) are coefficients for HPS.
(ii).

In all the previous examples, we recognized that dealing with HPS is more involved than working with ordinary series, where we only have to check that the final result is a convergent series “of the form” $\sum _{n=0}^{\infty }a_{n}(x-c)^{n}$. On the contrary, for HPS we have to control the following steps:

1. We have to check that the net $(a_{n\varepsilon })_{n,\varepsilon }$ defines coefficients for HPS (Definition 3(i)), i.e. that
  $$\begin{aligned} \forall ^{0}\varepsilon \,\forall n\in \mathbb {N}:\ |a_{n\varepsilon }|\le \rho _{\varepsilon }^{-nQ-R} \end{aligned}$$
  for some *Q*, $R\in \mathbb {N}_{>0}$. This allows us to talk about the radius of convergence $\textrm{rad}\left( a_{n}\right) _\textrm{c}$ and of the set of convergence  (Definition 8). Because of Theorem 9, this set is always non-trivial 2.14 but in general *is not an interval*, like the case of the exponential function clearly shows. This step already allows us to say that the HPS  is convergent, i.e. Definition 16, if .
2. At this point, we can study the set of convergence, e.g. to arrive at an explicit form . This depends mainly on three conditions:
  - For all $x\in C$, we must have a formal HPS (Definition 1) because this allows us to talk of any hyperfinite sum $\sum _{n=N}^{M}a_{n}(x-c)^{n}$ for *M*, . Here, the main step is to prove that the net $\left( \sum _{n=N_{\varepsilon }}^{M_{\varepsilon }}a_{n\varepsilon }(x_{\varepsilon }-c_{\varepsilon })^{n}\right) \in \mathbb {R}_{\rho }$.
  - For all $x\in C$, we have to check Definition 8. (iii), i.e. the equality: 2.15
  - Finally, we have to prove that for all representatives $x=[{\bar{x}}_{\varepsilon }]\in C$, all the derivatives $\frac{\textrm{d} ^{k}}{\textrm{d}{x}^{k}}\left( \sum _{n=0}^{+\infty }a_{n\varepsilon }({\bar{x}}_{\varepsilon }-c_{\varepsilon })^{n}\right)$ are $\rho$-moderate.
  - After the previous three steps, we get , and hence it remains to prove the opposite inclusion.
  See Corollary 25 for sufficiently general conditions under which only (2.15) suffices to prove that *x* lies in the set of convergence.

Note explicitly that we *never* formally defined what is a HPS: we have *formal HPS* (Definition 1), the notion of *coefficients for HPS* (Definition 3(i)), which always have a strictly positive *radius of convergence* (Definition 3(iii)) and a non trivial *set of convergence* (Definition 8 and Theorem 9), and finally *convergent HPS *(Definition 16).

### Topological properties of the set of convergence

The first consequence of our definition of convergent HPS Definition 16 and radius of convergence Definition 3, is the following

#### Lemma 17

Let  be a convergent HPS. If the following least upper bound exists2.16then $r\le \textrm{rad}\left( a_{n}\right) _\textrm{c}$.

#### Proof

In fact, if  is a convergent HPS, then , and hence $|{\bar{x}}_{\varepsilon }-c_{\varepsilon }|\le (\limsup _{n}\left| a_{n\varepsilon }\right| ^{1/n})^{-1}$ for all $\varepsilon$ small, i.e. $|{\bar{x}}-c|\le \textrm{rad}\left( a_{n}\right) _\textrm{c}$. $\square$

Note that from Example 14, we have that the least upper bound of2.17does not exist in , whereas Definition 3 yields the value $\textrm{rad}\left( \frac{1}{n!}\right) _\textrm{c}=+\infty$. Therefore, Definition 3 allows us to consider the exponential HPS even if the supremum of (2.17) does not exist. It remains an open problem whether $r=\textrm{rad}\left( a_{n}\right) _\textrm{c}$, at least if the least upper bound (2.16), or the corresponding sharp supremum, exists.

We now study absolute convergence of HPS, and sharply boundedness of the summands of a HPS. We first show that the hypersequence  of the terms of a HPS is sharply bounded:

#### Lemma 18

Let *x*, . If  is a convergent HPS, then2.18

#### Proof

We recall that because of the definition of formal HPS (Definition 1) and [25, Lemma 7] the term  is well-defined for all . Set ${\bar{x}}:=x-c$, i.e. without loss of generality we can assume $c=0$. Since  converges, from [25, Lemma 15] we have2.19Let us consider an arbitrary . From [21, Lemma 13], we have either $n\ge N$ or $n<_{L}N$ for some $L\subseteq _{0}I$. In the latter case, $|a_{n}{\bar{x}}^{n}|\le _{L}s:=\sum _{n=0}^{N-1}|a_{n}{\bar{x}}^{n}|<\max (s+1,1)=:K$. From [21, Lemma 7(iii)] and from (2.19), the claim follows. $\square$

The previous proof is essentially the generalization in our setting of the classical one, see e.g. [20]. However, property (2.18) does not allow us to apply the direct comparison test [25, Theorem 22]. Indeed, let us imagine that we only prove $\left| a_{n}x^{n}\right| <Kh^{n}$, with $h<1$, for all  and with *K* coming from (2.18); as we already explained in [25, Sec. 3.3], this would imply

$$\begin{aligned} \forall n\in \mathbb {N}\,\exists \varepsilon _{0n}\,\forall \varepsilon \le \varepsilon _{0n}:\ \left| a_{n\varepsilon }x_{\varepsilon }^{n}\right| \le K_{\varepsilon }h_{\varepsilon }^{n}, \end{aligned}$$

and the dependence of $\varepsilon _{0n}$ from $n\in \mathbb {N}$ is a problem in estimating inequalities of the form $\sum _{n=0}^{N_{\varepsilon }}\left| a_{n\varepsilon }x_{\varepsilon }^{n}\right| \le K_{\varepsilon }\sum _{n=0}^{N_{\varepsilon }}h_{\varepsilon }^{n}$, see [25]. A solution of this problem is to consider a uniform property of $n\in \mathbb {N}$ with respect to $\varepsilon$:

#### Definition 19

Let  and *x*, , then we say that $\left( a_{n}(x-c)^{n}\right) _{n\in \mathbb {N}}$ *is eventually* -*bounded in* , if there exist representatives $(a_{n})_{\textrm{c}}=\left[ a_{n\varepsilon }\right] _{{\textrm{c}}}$, $[x_{\varepsilon }]=x$, $[c_{\varepsilon }]=c$ such that2.20

#### Remark 20

(i) The adverb *eventually* clearly refers to the validity of the uniform inequality in (2.20) only for *n* sufficiently large.
(ii) If for $\varepsilon$ small, the series $\sum _{n=0}^{+\infty } \left| a_{n\varepsilon }(x_{\varepsilon }-c_{\varepsilon })^{n}\right| =:R_{\varepsilon }$ of absolute values terms converges to a $\rho$-moderate net, then (2.20) holds for $N=0$. This includes Example 11 of geometric hyperseries, Example 12 of a function with a flat point if both *x*, *c* are finite, and Example 14 of the exponential hyperseries at $c=0$ if *x* is finite.
(iii) In Example 15 of Dirac delta at $c=0$, if $|bx|\le 1$ (therefore, *x* is an infinitesimal number) we have $\left| \frac{\delta _{\varepsilon }^{(n)}(0)}{n!}x_{\varepsilon }^{n}\right| =\left| \frac{\mu ^{(n)}(0)}{n!}b_{\varepsilon }^{n+1}x_{\varepsilon }^{n}\right| \le b_{\varepsilon }$ for all $n\in \mathbb {N}$ such that $\left| \frac{\mu ^{(n)}(0)}{n!}\right| \le 1$. Therefore, $\left( \frac{\delta ^{(n)}(0)}{n!}x^{n}\right) _{n\in \mathbb {N}}$ is eventually -bounded in  if $|bx|\le 1$. If $x\gg 0$, i.e. $x\ge s\in \mathbb {R}_{>0}$, then $\left| \frac{\delta _{\varepsilon }^{(n)}(0)}{n!}x_{\varepsilon }^{n}\right| =\left| \frac{\mu ^{(n)}(0)}{n!}b_{\varepsilon }^{n+1}x_{\varepsilon }^{n} \right| \ge \left| \frac{\mu ^{(n)}(0)}{n!}\right| s^{n}b_{\varepsilon }^{n+1}$ and hence condition (2.20) does not hold for any  because $b\ge \textrm{d}\rho ^{-a}$ for some $a\in \mathbb {R}_{>0}$ (see e.g. [12, Sec. 3.0.2]).

The last example also shows that property (2.20) does not hold for all points . However, it always holds for any *c* if *x* is sufficiently near to *c*:

#### Lemma 21

Let  and , then there exists  such that for all $x\in B_{\sigma }(c)$, the sequence of summands $\left( a_{n}(x-c)^{n}\right) _{n\in \mathbb {N}}$ is eventually -bounded in .

#### Proof

Using the same notation as above, since , we have $\forall ^{0}\varepsilon \,\forall n\in \mathbb {N}:\ |a_{n\varepsilon }|\le \rho _{\varepsilon }^{-nQ-R}$. Therefore, for $\sigma :=\textrm{d}\rho ^{Q}$, we have $|a_{n\varepsilon }(x_{\varepsilon }-c_{\varepsilon })^{n}|\le \rho _{\varepsilon }^{-nQ-R}\rho _{\varepsilon }^{nQ}=\rho _{\varepsilon }^{-R}$. $\square$

The following result is a stronger version of the previous Lemma 18, and allows us to apply the dominated convergence test:

#### Lemma 22

Let , *x*, , and assume that $\left( a_{n}(x-c)^{n}\right) _{n\in \mathbb {N}}$ is eventually -bounded in , then2.21i.e. for all representatives $(a_{n})_{\textrm{c}}=\left[ a_{n\varepsilon }\right] _{{\textrm{c}}}$, $[x_{\varepsilon }]=x$, $[c_{\varepsilon }]=c$, $[K_{\varepsilon }]=K$, we have

2.22 $$\begin{aligned} \forall ^{0}\varepsilon \,\forall n\in \mathbb {N}:\ \left| a_{n\varepsilon }(x_{\varepsilon }-c_{\varepsilon })^{n}\right| <K_{\varepsilon }. \nonumber \\ \end{aligned}$$

Since  by Remark 5(ii), property (2.21) also shows that Definition 19 does not depend on the representatives involved.

#### Proof

It suffices to set $K:=R\vee \max _{n\le N}a_{n}$, where  and $N\in \mathbb {N}$ come from (2.20). $\square$

Even if the case of the exponential HPS (see Example 14) shows that in general the set of convergence is not an interval, it has very similar properties, at least if the gauge $\sigma$ is sufficiently small:

#### Theorem 23

Let $\sigma \le \rho ^{*}$ and  be a convergent HPS whose sequence of summands $\left( a_{n}({\bar{x}}-c)^{n}\right) _{n\in \mathbb {N}}$ is eventually -bounded in . Then for all $x\in B_{|{\bar{x}}-c|}(0)$ we have:

(i) The HPS converges absolutely at *x*, and hence uniformly on every functionally compact $K\Subset _{\textrm{f}}{\overline{B}}_{|{\bar{x}}-c|}(c)$;
(ii) $\left( a_{n}(x-c)^{n}\right) _{n\in \mathbb {N}}$ is eventually -bounded in .
(iii If $|{\hat{x}}-c|=|{\bar{x}}-c|$, then not necessarily  converges.

If , then:

(iv);
(v) *x* is a sharply interior point, i.e.  for some ;
(vi) is -convex, i.e. if also , then ;
(vii) The set of convergence  is *strongly connected*, i.e. it is not possible to write it as union of two non empty strongly disjoint sets, i.e. such that
  - *A*, , $$A\ne \emptyset \ne B$$,
  - $$\exists \sup (A)$$, $$\exists \inf (B)$$, $$\sup (A)\le \inf (B)$$,
  ,
  - $$\exists m\in \mathbb {N}:\ B_{\textrm{d}\rho ^{m}}(A) \cap B_{\textrm{d}\rho ^{m}}(B)=\emptyset $$.

#### Proof

Without loss of generality we can assume $c=0$. From [21, Lemma 5(ii)], we have either ${\bar{x}}=_{L}0$ or $|{\bar{x}}|>0$ for some $L\subseteq _{0}I$. The first case is actually impossible because $0\le |x|<|{\bar{x}}|=_{L}0$. We can hence work only in the latter case $|{\bar{x}}|>0$. From Lemma 22, we have $\forall ^{0}\varepsilon \,\forall n\in \mathbb {N}:\ |a_{n\varepsilon }{\bar{x}}_{\varepsilon }^{n}|\le K_{\varepsilon }$. Setting $h:=\left| \frac{x}{{\bar{x}}}\right|$, we have $h<1$ because $|x|\in B_{|{\bar{x}}|}(0)$, and

2.23 $$\begin{aligned} \forall ^{0}\varepsilon \,\forall n\in \mathbb {N}:\ |a_{n\varepsilon }x_{\varepsilon }^{n}|=\left| a_{n\varepsilon }{\bar{x}}_{\varepsilon }^{n}\right| .\left| \frac{x_{\varepsilon }}{{\bar{x}}_{\varepsilon }}\right| ^{n} <K_{\varepsilon }h_{\varepsilon }^{n}. \nonumber \\ \end{aligned}$$

Thereby, $\sum _{n=N}^{M}|a_{n}x^{n}|\le \sum _{n=N}^{M}Kh^{n}$ for all *N*, . By the direct comparison test [25, Theorem 22], the HPS  converges absolutely because  converges since $\sigma \le \rho ^{*}$ and $h<1$. Finally, [12, Theorem 74] yields that pointwise convergence implies uniform convergence on functionally compact sets, see [11]. This proves (i).

(ii): From (2.23) it follows that $\sum _{n=0}^{+\infty }|a_{n\varepsilon }x_{\varepsilon }^{n}|=:R_{\varepsilon }$ converges and is $\rho$-moderate. This implies condition (2.20).

For (iii), it suffices to consider that  converges (see [25, Sec. 3.6]) whereas  does not by [25, Theorem 18]. Note however, that for $x=1$, we have $|x|=\textrm{rad}\left( \frac{1}{n}\right) _\textrm{c}$ so that condition Definition 8(i) does not hold.

(iv): From the assumptions, $x\in B_{|{\bar{x}}-c|}(0)$, $|{\bar{x}}-c|<\textrm{rad}\left( a_{n}\right) _\textrm{c}$, and hence Definition 8(i) and Definition 8(iii follow. Note that Definition 8(ii) can be proved as above from (2.23). Finally, if $[{\hat{x}}_{\varepsilon }]=x$ and $k\in \mathbb {N}_{>0}$, we have

2.24 $$\begin{aligned} \frac{\textrm{d} ^{k}}{\textrm{d}{x}^{k}}\left( \sum _{n=0}^{+\infty }a_{n\varepsilon } {\hat{x}}_{\varepsilon }^{n}\right)&\le \sum _{n=k}^{+\infty }|a_{n\varepsilon }|k!{n \atopwithdelims ()k} \left| \frac{{\hat{x}}_{\varepsilon }}{{\bar{x}}_{\varepsilon }}\right| ^{n-k}|{\bar{x}}_{\varepsilon }|^{n-k}\nonumber \\&\le K_{\varepsilon }|{\bar{x}}_{\varepsilon }|^{-k}\sum _{n=k}^{+\infty }k!{n \atopwithdelims ()k}\left| \frac{{\hat{x}}_{\varepsilon }}{{\bar{x}}_{\varepsilon }}\right| ^{n-k}\in \mathbb {R}_{\rho }, \nonumber \\ \end{aligned}$$

where we used Lemma 22, and hence Definition 8(iv) also holds.

(v): For $s:=|{\bar{x}}|-|x|>0$ and ${\hat{x}}\in B_{s}(x)$, we have $|{\hat{x}}|\le |{\hat{x}}-x|+|x|<s+|x|=|{\bar{x}}|$, and hence  from (iv).

(vi): Setting ${\hat{x}}:=y+t({\bar{x}}-y)$, we have $y\le {\hat{x}}\le {\bar{x}}$. We can use trichotomy law [21, Lemma 7(iii)] to distinguish the cases $y=_{L}0$ or $y>_{L}0$ or $y<_{L}0$ for $L\subseteq _{0}I$. The latter has to be subdivided into the sub-cases ${\hat{x}}>_{M}0$ or ${\hat{x}}=_{M}0$ or ${\hat{x}}<_{M}0$ with $M\subseteq _{0}L$, i.e. using [21, Lemma 7(iii)] for the ring . Finally, the latter of these sub-cases has to be further subdivided into ${\hat{x}}>_{K}y$ or ${\hat{x}}<_{K}y$ or ${\hat{x}}=_{K}y$ with $K\subseteq _{0}M$. In all these cases (clearly, some of these subcases cannot hold simultaneously) we can prove Definition 8 in the corresponding co-final set.

(vii): By contradiction, if $a\in A$ and $b\in B$, then $x:=\frac{1}{2}\left( \sup (A)+\inf (B)\right)$ lies in the segment  by (vi). But property $B_{\textrm{d}\rho ^{m}}(A)\cap B_{\textrm{d}\rho ^{m}}(B)$ implies that $\sup (A)<\inf (B)$ and hence . $\square$

In spite of Theorem 23(v), it remains open the problem whether the set of convergence is always a sharply open set or not. Using the previous theorem, this problem depends, for each point *x* in the set of convergence, on the existence of a point ${\bar{x}}$ satisfying assumptions of Theorem 23. However, in the case , Remark 20 (iv) shows that $\left( \frac{\delta ^{(n)}(0)}{n!}x^{n}\right) _{n\in \mathbb {N}}$ is not eventually -bounded in , so that such a point ${\bar{x}}$ in this case does not exist and Theorem 23 cannot be applied.

#### Corollary 24

Let $\sigma \le \rho ^{*}$ and let *R* be the set of all the numbers of the form $s=|{\bar{x}}-c|$ for some  satisfying:

(i) is a convergent HPS,
(ii) $(a_{n}({\bar{x}}-c)^{n})_{n\in \mathbb {N}}$ is eventually -bounded in .

If , then , the HPS  converges absolutely for all $x\in B_{r}(c)$ and uniformly on every functionally compact $K\Subset _{\textrm{f}}{\overline{B}}_{r}(c)$.

#### Proof

Without loss of generality, we assume $c=0$, and let $x\in B_{r}(c)$. Since $|x|<r$, by the definition of sharp supremum, (see [21]) there exist $s=\left| {\bar{x}}\right|$ such that $|x|<\left| {\bar{x}}\right| \le r$ and such that (i) and (ii) hold. The conclusions then follow by Theorem 23. $\square$

Property Theorem 23(iv) can also be written as a characterization of the set of convergence:

#### Corollary 25

Let $\sigma \le \rho ^{*}$, ,  such that  is a convergent HPS whose sequence of summands $\left( a_{n}({\bar{x}}-c)^{n}\right) _{n\in \mathbb {N}}$ is eventually -bounded in . If $x\in B_{|{\bar{x}}-c|}(0)$, then  if and only if

### Algebraic properties of hyper-power series

In this section, we extend to HPS the classical results concerning algebraic operations and composition of power series.

#### Theorem 26

Assume that  and  are two convergent HPS, then:

(i) For all , the product  is a convergent HPS with $\textrm{rad}\left( ra_{n}\right) _\textrm{c}\ge \textrm{rad}\left( a_{n}\right) _\textrm{c}$, and 2.25
(ii) The sum of these HPS is a convergent HPS with
  $$\begin{aligned} \textrm{rad}\left( a_{n}+b_{n}\right) _\textrm{c}\ge \min (\textrm{rad}\left( a_{n}\right) _\textrm{c},\textrm{rad}\left( b_{n}\right) _\textrm{c}), \end{aligned}$$
  and 2.26
(iii) For all ${\bar{x}}\in B_{|x-c|}(c)$, the product of these HPS converges to their Cauchy product: 2.27 which is still a convergent HPS with radius of convergence greater or equal to $\min (\textrm{rad}\left( a_{n}\right) _\textrm{c},\textrm{rad}\left( b_{n}\right) _\textrm{c})$.
(iv) Let $\left[ a_{n\varepsilon }\right] _{\text {c}}=(a_{n})_{\textrm{c}}$ and $\left[ b_{n\varepsilon }\right] _{\text {c}}=(b_{n})_{\textrm{c}}$ be representatives of the coefficients of the given HPS. Assume that  is invertible, and recursively define (for $\varepsilon$ small) $d_{0\varepsilon }:=\frac{a_{0\varepsilon }}{b_{0\varepsilon }}$,
  2.28 $$\begin{aligned} d_{n\varepsilon }:=\frac{1}{b_{0\varepsilon }}\left( a_{n\varepsilon }-\sum _{l=1}^{n}b_{l\varepsilon }d_{n-l,\varepsilon }\right) \qquad \forall n\in \mathbb {N}_{>0}. \end{aligned}$$
  Then coefficients  define a convergent HPS with radius of convergence greater or equal to $\min (\textrm{rad}\left( a_{n}\right) _\textrm{c},\textrm{rad}\left( b_{n}\right) _\textrm{c})$ such that for all ${\bar{x}}\in B_{|x-c|}(c)$, if  is invertible, then 2.29

#### Proof

Equalities (2.25) and (2.26) follow directly from analogous properties of convergent hyperlimits, i.e. [21, Sec. 5.2]. All the inequalities concerning the radius of convergence can be proved in the same way from analogous results of the classical theory, because of Definition (iii) 3. For example, from Definition 8(iii) we have that both the ordinary series $\sum _{n=0}^{+\infty }a_{n\varepsilon }(x_{\varepsilon }-c_{\varepsilon })^{n}$ and $\sum _{n=0}^{+\infty }b_{n\varepsilon }(x_{\varepsilon }-c_{\varepsilon })^{n}$ converge. Thereby, their sum $\sum _{n=0}^{+\infty }\left( a_{n\varepsilon }+b_{n\varepsilon }\right) (x_{\varepsilon }-c_{\varepsilon })^{n}$ converges with radius $\textrm{rad}\left( a_{n}+b_{n}\right) _{\textrm{c}{\varepsilon }}\ge \min \left( \textrm{rad}\left( a_{n}\right) _{\textrm{c}{\varepsilon }},\textrm{rad}\left( b_{n}\right) _{\textrm{c}{\varepsilon }}\right)$. To prove (2.27) (assuming that ${\bar{x}}$ lies in the convergence set of the product HPS, see below), from Lemma 23 we have that both the series converge absolutely because ${\bar{x}}\in B_{|x-c|}(c)$. We can hence apply the generalization of Mertens’ theorem to hyperseries (see [25, Theorem 37]).

To complete the proof of (iii), we start by showing that the representatives of the product $\left( \sum _{k=0}^{n}a_{k\varepsilon }b_{n-k,\varepsilon }\right) _{n,\varepsilon }$ defines coefficients for an HPS. Let $(a_{n})_{{\textrm{c}}}=\left[ a_{n\varepsilon }\right] _{{\textrm{c}}}$, , so that:

2.30 $$\begin{aligned} \exists Q_{1},R_{1}\in \mathbb {N}\,\forall ^{0}\varepsilon \,\forall n\in \mathbb {N}:\ \left| a_{n\varepsilon }\right| \le \rho _{\varepsilon }^{-nQ_{1}-R_{1}}. \end{aligned}$$

2.31 $$\begin{aligned} \exists Q_{2},R_{2}\in \mathbb {N}\,\forall ^{0}\varepsilon \,\forall n\in \mathbb {N}:\ \left| b_{n\varepsilon }\right| \le \rho _{\varepsilon }^{-nQ_{2}-R_{2}}. \nonumber \\ \end{aligned}$$

Without loss of generality we can assume $Q_{2}>Q_{1}$. We have

2.32 $$\begin{aligned} \left| \sum _{k=0}^{n}a_{k\varepsilon }b_{n-k,\varepsilon }\right|&\le \sum _{k=0}^{n}|a_{k\varepsilon }||b_{n-k,\varepsilon }|\nonumber \\&\le \sum _{k=0}^{n}\rho _{\varepsilon }^{-kQ_{1}-R_{1}}.\rho _{\varepsilon } ^{-(n-k)Q_{2}-R_{2}}\nonumber \\&\le \sum _{k=0}^{n}\rho _{\varepsilon }^{-kQ_{1}+kQ_{2}-nQ_{2}-R_{1} -R_{2}}. \nonumber \\ \end{aligned}$$

We have $\rho _{\varepsilon }^{Q_{2}-Q_{1}}<1$ because $Q_{2}>Q_{1}$, and hence

$$\begin{aligned} \left| \sum _{k=0}^{n}a_{k\varepsilon }b_{n-k,\varepsilon }\right| \le \frac{\rho _{\varepsilon } ^{-nQ_{2}-R_{1}-R_{2}}}{1-\rho _{\varepsilon }^{-Q_{1}+Q_{2}}}\le \rho _{\varepsilon }^{-nQ-R}, \end{aligned}$$

where $R:=R_{1}+R_{2}$ and for a suitable $Q\in \mathbb {N}$ (that can be chosen uniformly with respect to $n\in \mathbb {N}$). Thereby, the product HPS has well-defined coefficients and hence a suitable set of convergence.

Now, we want to show that ${\bar{x}}$ lies in this set of convergence. Since Definition 8(i) clearly holds and Definition 8(iii) follows from Mertens’ Theorem (both [25, Theorem 37] and the classical version), it remains to prove that we actually have a formal HPS (Definition 8(ii)) and moderateness of derivatives (Definition 8(iv)). The latter follows by the general Leibniz rule for the *k*-th derivative of a product. For the former one, without loss of generality we can assume $c=0$; let $\left( M_{\varepsilon }\right)$, $\left( N_{\varepsilon }\right) \in \mathbb {N}_{\sigma }$, then for suitable $\left( {\bar{M}}_{\varepsilon }\right)$, $\left( {\hat{M}}_{\varepsilon }\right) \in \mathbb {N}_{\sigma }$ and $\left( {\bar{N}}_{\varepsilon }\right)$, $\left( {\hat{N}}_{\varepsilon }\right) \in \mathbb {N}_{\sigma }$ such that $M_{\varepsilon }={\bar{M}}_{\varepsilon }+{\hat{M}}_{\varepsilon }$ and $N_{\varepsilon }={\bar{N}}_{\varepsilon }+{\hat{N}}_{\varepsilon }$, we have

2.33 $$\begin{aligned} \left( \sum _{n=N_{\varepsilon }}^{M_{\varepsilon }}\sum _{k=0}^{n}a_{n\varepsilon }b_{n-k,\varepsilon } {\hat{x}}_{\varepsilon }^{n}\right) =\left( \sum _{n={\bar{N}}_{\varepsilon }}^{{\bar{M}}_{\varepsilon }} a_{n\varepsilon }{\hat{x}}_{\varepsilon }^{n}\right) \cdot \left( \sum _{n={\hat{N}}_{\varepsilon }}^ {{\hat{M}}_{\varepsilon }}b_{n,\varepsilon }{\hat{x}}_{\varepsilon }^{n}\right) , \end{aligned}$$

and thereby Definition 8(ii) follows.

(iv): To prove that , without loss of generality, we can assume in (2.30) and (2.31) that $Q_{1}=Q_{2}=:{\hat{Q}}>R_{1}=R_{2}=:{\hat{R}}$ and ${\hat{Q}}>0$. By induction on $n\in \mathbb {N}$, we want to prove that

2.34 $$\begin{aligned} \forall ^{0}\varepsilon \,\forall n\in \mathbb {N}:\ \left| d_{n\varepsilon }\right| \le \rho _{\varepsilon }^{-n{\hat{Q}}-{\hat{Q}}}. \nonumber \\ \end{aligned}$$

For $n=0$, we have $\left| d_{0\varepsilon }\right| =\left| \frac{a_{0\varepsilon }}{b_{0\varepsilon }} \right| \le \rho _{\varepsilon }^{-{\hat{R}}+{\hat{R}}}\le \rho _{\varepsilon }^{-{\hat{Q}}}$ for all $\varepsilon$ because ${\hat{Q}}>0$. For the inductive step, we assume (2.34) and use the recursive definition (2.28):

$$\begin{aligned} \left| d_{n+1,\varepsilon }\right|&\le \left| \frac{a_{n+1,\varepsilon }}{b_{0\varepsilon }}\right| +\left| \frac{\sum _{l=1}^{n+1}b_{l\varepsilon }d_{n-l,\varepsilon }}{b_{0\varepsilon }}\right| \\&\le \rho _{\varepsilon }^{-(n+1){\hat{Q}}-{\hat{R}}}\cdot \rho _{\varepsilon }^{{\hat{R}}} +\sum _{l=1}^{n+1}\rho _{\varepsilon }^{-l{\hat{Q}}-{\hat{R}}}\ cdot\rho _{\varepsilon }^{-(n-l){\hat{Q}}-{\hat{Q}}}\cdot \rho _{\varepsilon }^{{\hat{R}}}\\&=\rho _{\varepsilon }^{-n{\hat{Q}}-{\hat{Q}}}+\rho _{\varepsilon }^{-n{\hat{Q}} -{\hat{Q}}}\le 2\rho _{\varepsilon }^{-n{\hat{Q}}-{\hat{Q}}}. \end{aligned}$$

We have $2\rho _{\varepsilon }^{-n{\hat{Q}}-{\hat{Q}}}\le \rho _{\varepsilon }^{-(n+1){\hat{Q}}-{\hat{Q}}}$ if and only if $2\le \rho _{\varepsilon }^{-{\hat{Q}}}$, which holds for $\varepsilon$ small (independently by *n*).

Finally, equality (2.29) can be proved as we did above for the product because ${\bar{x}}\in B_{|x-c|}(c)$ and2.35From this equality, it also follows Definition 16(ii) because the product of an invertible non-moderate net (on a co-final set) by a moderate net cannot yield a moderate net. Finally, as above, moderateness of derivatives follows from Mertens’ theorem and the *k*-th derivative of the quotient. $\square$

The following theorem concerns the composition of HPS:

#### Theorem 27

Let $(a_{n})_{{\textrm{c}}}=\left[ a_{n\varepsilon }\right] _{{\textrm{c}}}$,  be coefficients for HPS. SetSet

$$\begin{aligned} c_{0\varepsilon }&:=a_{0\varepsilon }\\ c_{n\varepsilon }&:=\sum _{k=0}^{+\infty }a_{k\varepsilon }\sum _{m_{1}+\cdots +m_{k}=n}b_{m_{1}\varepsilon }\cdot \cdots \cdot b_{m_{k}\varepsilon }\qquad \forall n\in \mathbb {N}_{>0}. \end{aligned}$$

If  and , thenis a convergent HPS.

#### Proof

Since $\left[ a_{n\varepsilon }\right] _{c}$, , we can assume that both (2.2) and (2.3) hold with ${\hat{Q}}=Q_{1}=Q_{2}>0$ and ${\hat{R}}=R_{1}=R_{2}>0$. We have

$$\begin{aligned} \left| \sum _{k=0}^{n}a_{k\varepsilon }\sum _{m_{1}+\cdots +m_{k}=n}b_{m_{1}\varepsilon }\right.&\left. \cdot \cdots \cdot b_{m_{k}\varepsilon }{\sum _{k=0}^{n}}\right| \le \sum _{k=0}^{n}|a_{k\varepsilon }|\sum _{m_{1}+\cdots +m_{k}=n}|b_{m_{1}\varepsilon } |\cdot \cdots \cdot |b_{m_{k}\varepsilon }|\\&\le \sum _{k=0}^{n}\rho _{\varepsilon }^{-k{\hat{Q}}-{\hat{R}}}\sum _{m_{1} +\cdots +m_{k}=n}\rho _{\varepsilon }^{-m_{1}{\hat{Q}}-{\hat{R}}}\cdot \cdots \cdot \rho _{\varepsilon }^{-m_{k}{\hat{Q}}-{\hat{R}}}\\&=\sum _{k=0}^{n}\rho _{\varepsilon }^{-k{\hat{Q}}-{\hat{R}}}\sum _{m_{1} +\cdots +m_{k}=n}\rho _{\varepsilon }^{-n{\hat{Q}}-k{\hat{R}}}\\&=\rho _{\varepsilon }^{-{\hat{R}}}+\sum _{k=1}^{n}\rho _{\varepsilon }^{-k{\hat{Q}} -{\hat{R}}}\sum _{m_{1}+\cdots +m_{k}=n}\rho _{\varepsilon }^{-n{\hat{Q}}-k{\hat{R}}}\\&=\rho _{\varepsilon }^{-{\hat{R}}}+\sum _{k=1}^{n}\rho _{\varepsilon }^{-k{\hat{Q}} -{\hat{R}}-n{\hat{Q}}-k{\hat{R}}}{n+k-1 \atopwithdelims ()k-1}\\&\le \rho _{\varepsilon }^{-{\hat{R}}}+2^{2n}\rho _{\varepsilon }^{-{\hat{R}}-n{\hat{Q}}} \cdot \frac{1-\rho _{\varepsilon }^{-(n+1)({\hat{Q}} +{\hat{R}})}}{1-\rho _{\varepsilon }^{-{\hat{Q}}-{\hat{R}}}}=:[*]. \end{aligned}$$

For $\varepsilon$ small, we have $\frac{4}{\rho _{\varepsilon ^{-1}}}\le 1$, hence $\frac{2^{2n}}{\rho _{\varepsilon }^{-n}}\le 1$ for the same $\varepsilon$ and for all $n\in \mathbb {N}$. Now, take $\varepsilon$ small so that also $\frac{1}{1-\rho _{\varepsilon }^{-{\hat{Q}}-{\hat{R}}}}\le 1$, and $\frac{1}{\rho _{\varepsilon }^{-1}}\le \frac{1}{3}$. We hence have

$$\begin{aligned} [*]\le \rho _{\varepsilon }^{-{\hat{R}}}+\rho _{\varepsilon }^{-n{\hat{Q}}-{\hat{R}}-n} +\rho _{\varepsilon }^{-2n{\hat{Q}}-n{\hat{R}}-2{\hat{R}}-n}. \end{aligned}$$

Since

$$\begin{aligned} \frac{\rho _{\varepsilon }^{-{\hat{R}}}}{\rho _{\varepsilon }^{-n(2{\hat{Q}}+{\hat{R}}+1) -2{\hat{R}}-1}}&\le \frac{1}{\rho _{\varepsilon }^{-1}}\le \frac{1}{3}\\ \frac{\rho _{\varepsilon }^{-n{\hat{Q}}-{\hat{R}}-n}}{\rho _{\varepsilon }^{-n(2{\hat{Q}} +{\hat{R}}+1)-2{\hat{R}}-1}}&\le \frac{1}{\rho _{\varepsilon }^{-1}}\le \frac{1}{3}\\ \frac{\rho _{\varepsilon }^{-2n{\hat{Q}}-n{\hat{R}}-2{\hat{R}}-n}}{\rho _{\varepsilon }^{-n(2{\hat{Q}}+{\hat{R}}+1)-2{\hat{R}}-1}}&\le \frac{1}{\rho _{\varepsilon }^{-1}}\le \frac{1}{3}, \end{aligned}$$

we finally get

$$\begin{aligned} \forall ^{0}\varepsilon \,\forall n\in \mathbb {N}:\ \left| \sum _{k=0}^{n}a_{k\varepsilon }\sum _{m_{1}+\cdots +m_{k}=n}b_{m_{1}\varepsilon }\cdot \cdots \cdot b_{m_{k}\varepsilon }\right| \le \rho _{\varepsilon }^{-n(2{\hat{Q}}+{\hat{R}}+1)-2{\hat{R}}-1}, \end{aligned}$$

which proves that $(c_{n\varepsilon })_{n,\varepsilon }$ defines coefficients for an HPS.

To prove that , we can proceed as follows: Definition 8(i) can be proved like in the classical case; Definition 8(ii) is a consequence of composition of polynomials if $M_{\varepsilon }<+\infty$ or it can be proved proceeding like in the case of composition of GSF if $M_{\varepsilon }=+\infty$: Definition 8(iii) and Definition 8(iv) can be proved like for GSF (see [12] and Theorem 28 below). $\square$

## Generalized real analytic functions and their calculus

A direct consequence of Definition 8 of set of convergence is the following

### Theorem 28

Let  and . Setfor all . Then  is a GSF defined by $\left( v_{\varepsilon }\right)$.

Before defining the notion of GRAF, we need to prove that the derived HPS has the same set of convergence of the original HPS:

### Theorem 29

Assume $\sigma \le \rho ^{*}$,  and . Then the set of convergence of the derived series  is the same as the set of convergence of the original HPS . Thereby, recursively, all the derivatives have the same set of convergence of the original HPS and define a GSF.

### Proof

By Definition 3(iii) of radius of convergence and the classical theory, we have

$$\begin{aligned} \textrm{rad}\left( a_{n}\right) _{\textrm{c}{\varepsilon }}&=\left( \limsup _{n\rightarrow +\infty }\left| a_{n\varepsilon }\right| ^{1/n}\right) ^{-1}=\left( \limsup _{n\rightarrow +\infty }\left| (n+1)a_{n+1,\varepsilon }\right| ^{1/n+1}\right) ^{-1}\\&=\textrm{rad}\left( (n+1)a_{n+1}\right) _{\textrm{c}{\varepsilon }}, \end{aligned}$$

so Definition 8(i) for the original HPS and the derived one are equivalent. From the condition  and $\sigma \le \rho ^{*}$, in the usual way it follows that . Vice versa, from $(n+1)\left| a_{n+1,\varepsilon }\right| \ge \left| a_{n+1,\varepsilon }\right|$ the opposite implication follows. The condition Definition 8(iv) about moderateness of derivatives for the original HPS clearly implies the analogue condition for the derived one. For the opposite inclusion, we can distinguish the case $x=_{\text {s}}c$ or $|x-c|>0$, the former one being trivial. We have

$$\begin{aligned} \left| \sum _{n=1}^{+\infty }a_{n\varepsilon }n(x_{\varepsilon }-c_{\varepsilon })^{n-1}\right|&=|x_{\varepsilon }-c_{\varepsilon }|^{-1}\left| \sum _{n=1}^{+\infty }a_{n\varepsilon }n(x_{\varepsilon }-c_{\varepsilon })^{n}\right| \\&\ge |x_{\varepsilon }-c_{\varepsilon }|^{-1}\left| \sum _{n=0}^{+\infty }a_{n\varepsilon }(x_{\varepsilon }-c_{\varepsilon })^{n}\right| , \end{aligned}$$

so that also the net $\left( \sum _{n=0}^{+\infty }a_{n\varepsilon }(x_{\varepsilon }-c_{\varepsilon })^{n}\right) \in \mathbb {R}_{\rho }$ if the derivative is moderate. $\square$

Theorem 28 motivates the following definition:

### Definition 30

Let $\sigma \le \rho ^{*}$ and *U* be a sharply open set of , then we say that *f* *is a GRAF on* *U* (with respect to $\rho$, $\sigma$), and we write  if  and for all $c\in U$ we can find ,  such that

(i),
(ii) for all $x\in (c-s,c+s)$.

Moreover, we say that  *is an entire function* (with respect to $\rho$, $\sigma$) if we can find  and  such that

(iii),
(iv) for all .

We also say that *f* *is entire at* *c* if (iii) and (iv) hold.

### Example 31

- Clearly, if , , and we set , then *f* is a GRAF on the interior points of the set of convergence . Vice versa, if , then *U* is contained in the union of all the sharp interior sets , because of condition (i).
- Example 15 shows that Dirac $\delta$ is entire at 0 but it is not at any  such that $\left| c\right| \ge s$ for some $s\in \mathbb {R}_{>0}$.
- Example 12 of a function *f* with a flat point shows that *f* is a GRAF, but if $c=0$, then  satisfying condition (i) is infinitesimal, whereas if $c\gg 0$, then $s\gg 0$ is finite, and these two types of set of convergence are always disjoint.

### Corollary 32

Let $\sigma \le \rho ^{*}$,  be a sharply open set and , then also  and it can be computed with the derived HPS i.e. .

Because of our definition Definition 8 of set of convergence, several classical results can be simply translated in our setting considering the real analytic function that defines a given GRAF.

### Theorem 33

Let $\sigma \le \rho ^{*}$, , , and set  for all interior points , then $a_{k}=\frac{f^{(k)}(c)}{k!}$ for all $k\in \mathbb {N}$.

### Proof

From Corollary 32, we have $f^{(k)}(x)=\left[ \sum _{n=k}^{\infty }a_{n\varepsilon }k!{n \atopwithdelims ()k}(x_{\varepsilon }-c_{\varepsilon })^{n-k}\right]$ for all the interior points . For $x=c$ (which is always a sharply interior point because of Theorem 9) this yields the conclusion. $\square$

### Corollary 34

Let $\sigma \le \rho ^{*}$, *U* be a sharply open set of , and . Then for all $c\in U$ the Taylor coefficients .

The definition of 1-dimensional integral of GSF by using primitives, allows us to get a simple proof of the term by term integration of GRAF:

### Theorem 35

In the assumptions of the previous theorem, setfor all the interior points . Then $F(x)=\int _{c}^{x}f(x)\,\textrm{d}{x}$ and *F* is a GRAF on the interior points of .

### Proof

The proof that  is a convergent HPS with the same set of convergence of *f* can be done as in Theorem 29, and hence *F* is a GRAF on the interior points of . The remaining part of the proof is straightforward by using Corollary 32, so that $F'(x)=f\left( x\right)$ and $F(c)=0$. and using [12, Theorem 42, Definition 43]. $\square$

We close this section by first noting that, differently with respect to the classical theory, if  for all , and we take another point , we do not have that ; in fact for $c={\bar{c}}$ this would yield the false equality . On the other hand, in the following result we show that :

### Theorem 36

In the assumptions of Theorem 33, if , then .

### Proof

In fact, since , we haveThereby, if , we haveTherefore, the usual proof, see e.g. [20], yields

$$\begin{aligned} \sum _{n=0}^{+\infty }\frac{(x_{\varepsilon }-{\bar{c}}_{\varepsilon })^{n}}{n!}\sum _{m\ge n}^{+\infty }\frac{f_{\varepsilon }^{(m)}(c_{\varepsilon })}{m!}n!{m \atopwithdelims ()n}(x_{\varepsilon }-c_{\varepsilon })^{m-n}=\sum _{n=0}^{+\infty } \frac{f_{\varepsilon }^{(n)}(c)}{n!}(x_{\varepsilon }-c_{\varepsilon })^{n} \end{aligned}$$

and hence , which implies the conclusion. $\square$

## Characterization of generalized real analytic functions, inversion and identity principle

The classical characterization of real analytic functions by the growth rate of the derivatives establishes a difference between GRAF and Colombeau real analytic functions:

### Theorem 37

Let $\sigma \le \rho ^{*}$, *U* be a sharply open set of , and  be a GSF defined by the net $(f_{\varepsilon })$. Then  if and only if for each $c\in U$ there exist $s=[s_{\varepsilon }]$, $C=\left[ C_{\varepsilon }\right]$,  such that $B_{s}(c)\subseteq U$ and

4.1 $$\begin{aligned} \forall [x_{\varepsilon }]\in B_{s}(c)\,\forall ^{0}\varepsilon \,\forall n\in \mathbb {N}:\ \left| f_{\varepsilon }^{(n)}(x_{\varepsilon })\right| \le C_{\varepsilon }\frac{n!}{R_{\varepsilon }^{n}}. \end{aligned}$$

### Proof

We prove that condition (4.1) is necessary. For $c\in U$, we have  for all $x\in (c-{\bar{s}},c+{\bar{s}})$ for some ${\bar{s}}>0$ from Definition 30 and Theorem 33. We first note that condition (4.1) can also be formulated as an inequality in  and as such it does not depend on the representatives involved. Therefore, from Theorem 28 and Theorem 33, without loss of generality, we can assume that the given net $(f_{\varepsilon })$ is of real analytic functions satisfying $f_{\varepsilon }(x)=\sum _{n=0}^{+\infty }\frac{f_{\varepsilon }^{(n)}(c_{\varepsilon })}{n!}(x-c_{\varepsilon })^{n}$ for all $x\in (c_{\varepsilon }-\textrm{rad}\left( a_{n}\right) _{\textrm{c}{\varepsilon }},c_{\varepsilon }+\textrm{rad}\left( a_{n}\right) _{\textrm{c}{\varepsilon }})$. From Lemma 21, locally the Taylor summands $\left( \frac{f^{(n)}(c)}{n!}({\bar{x}}-c)^{n}\right) _{n\in \mathbb {N}}$ are eventually -bounded in  if ${\bar{x}}$ is sufficiently near to $c=[c_{\varepsilon }]$, i.e. there exists  such that for each ${\bar{x}}=[{\bar{x}}_{\varepsilon }]\in B_{\sigma }(c)$ we have

4.2 $$\begin{aligned} \forall ^{0}\varepsilon \,\forall j\in \mathbb {N}:\ \left| \frac{f_{\varepsilon }^{(j)}(c_{\varepsilon })}{j!}({\bar{x}}_{\varepsilon }-c_{\varepsilon })^{j}\right| \le K_{\varepsilon }, \nonumber \\ \end{aligned}$$

for some . Set $s:=\frac{1}{2}\min (\sigma ,{\bar{s}})\in \mathbb {R}_{>0}$ and $S:=|{\bar{x}}-c|$, where ${\bar{x}}$ is any point such that $s<|{\bar{x}}-c|<\sigma$, so that $0<\frac{s}{S}<1$ and from (4.2) we obtain

4.3 $$\begin{aligned} \forall ^{0}\varepsilon \,\forall j\in \mathbb {N}:\ \left| f_{\varepsilon }^{(j)}(c_{\varepsilon })\right| \le K_{\varepsilon }\frac{j!}{S_{\varepsilon }^{j}}. \nonumber \\ \end{aligned}$$

For each $[x_{\varepsilon }]\in B_{s}(c)$, we have

$$\begin{aligned} f_{\varepsilon }^{(n)}(x_{\varepsilon })&=\sum _{j=n}^{+\infty }\frac{f_{\varepsilon }^{(j)}(c_{\varepsilon })}{j!}n!{j \atopwithdelims ()n}(x_{\varepsilon }-c_{\varepsilon })^{j-n}, \end{aligned}$$

and hence from (4.3):

$$\begin{aligned} \left| \frac{f_{\varepsilon }^{(n)}(x_{\varepsilon })}{n!}\right|&\le \sum _{j=n}^{+\infty }K_{\varepsilon }{j \atopwithdelims ()n}\frac{|x_{\varepsilon } -c_{\varepsilon }|^{j-n}}{S_{\varepsilon }^{j}}\\&\le \frac{K_{\varepsilon }}{S_{\varepsilon }^{n}}\sum _{j=n} ^{\infty }{j \atopwithdelims ()n}\left( \frac{s_{\varepsilon }}{S_{\varepsilon }}\right) ^{j-n}\\&=\frac{K_{\varepsilon }}{S_{\varepsilon }^{n}}\cdot \frac{1}{\left( 1-\frac{s_{\varepsilon }}{S_{\varepsilon }}\right) ^{n+1}}=\frac{K_{\varepsilon }}{\left( 1-\frac{s_{\varepsilon }}{S_{\varepsilon }}\right) } \cdot \frac{1}{\left( S_{\varepsilon }\left( 1-\frac{s_{\varepsilon }}{S_{\varepsilon }}\right) \right) ^{n}}, \end{aligned}$$

which is our claim for $C:=\frac{K}{1-\frac{s}{S}}$ and $R:=S\left( 1-\frac{s}{S}\right)$. Note that, differently with respect to the case of Colombeau real analytic functions [23], not necessarily the constant $\frac{1}{R}$ is finite, e.g. if $s\approx S$.

We now prove that the condition is sufficient. Let $c=[c_{\varepsilon }]\in U$ and $s=[s_{\varepsilon }]$, $C=[C_{\varepsilon }]$,  be the constants satisfying (4.1). Set ${\bar{s}}:=\frac{1}{2}\min (s,R,\textrm{rad}\left( \frac{f^{(n)}(c)}{n!}\right) _\textrm{c})$ and take $x\in B_{{\bar{s}}}(c)$. We first prove the equality . Let , with $N_{\varepsilon }\in \mathbb {N}$. For all $\varepsilon$, from Taylor’s formula for the smooth $f_{\varepsilon }$, we have

$$\begin{aligned} \left| \sum _{n=0}^{N}\frac{f^{(n)}(c)}{n!}(x-c)^{n}-f(x)\right| =\left[ \left| \frac{f_{\varepsilon }^{(N_{\varepsilon }+1)}(\xi _{\varepsilon })}{(N_{\varepsilon }+1)!} (x_{\varepsilon }-c_{\varepsilon })^{N_{\varepsilon }+1}\right| \right] \end{aligned}$$

for some $t_{\varepsilon }\in [0,1]_{\mathbb {R}}$ and for $\xi _{\varepsilon }:=(1-t_{\varepsilon })c_{\varepsilon }+t_{\varepsilon }x_{\varepsilon }$. Since $\left| \xi _{\varepsilon }-c_{\varepsilon }\right| =t_{\varepsilon }\left| x_{\varepsilon } -c_{\varepsilon }\right|<{\bar{s}}_{\varepsilon }<s_{\varepsilon }$, we can apply (4.1) and get $\forall ^{0}\varepsilon \,\forall n\in \mathbb {N}:\ \left| \frac{f_{\varepsilon }^{(n)}(\xi _{\varepsilon })}{n!}\right| \le \frac{C_{\varepsilon }}{R_{\varepsilon }^{n}}$. Thereby, for these small $\varepsilon$ and for $n=N_{\varepsilon }+1$ we obtain

$$\begin{aligned} \left| \sum _{n=0}^{N}\frac{f^{(n)}(c)}{n!}(x-c)^{n}-f(x)\right| \le C\left( \frac{{\bar{s}}}{R}\right) ^{N+1}, \end{aligned}$$

and hence the claim follows by . Now, we prove that . In fact, once again from (4.1) we havebecause ${\bar{s}}<R$ and hence  converges. Finally, take ${\bar{x}}\in B_{{\bar{s}}}(c)$ such that $|x-c|<|{\bar{x}}-c|$. As above, we can prove that  converges; moreover from (4.1) we also have $\forall ^{0}\varepsilon \,\forall n\in \mathbb {N}:\ \left| \frac{f_{\varepsilon }^{(n)}(c_{\varepsilon })}{n!}({\bar{x}}_{\varepsilon }-c_{\varepsilon })^{n}\right| \le C_{\varepsilon }\left( \frac{{\bar{s}}_{\varepsilon }}{R_{\varepsilon }}\right) ^{n} \le \frac{C_{\varepsilon }}{1-\frac{{\bar{s}}_{\varepsilon }}{R_{\varepsilon }}}$. This proves that $\left( \frac{f^{(n)}(c)}{n!}({\bar{x}}-c)^{n}\right) _{n\in \mathbb {N}}$ is eventually -bounded in  and hence  by Corollary 25. $\square$

As we have already noted in this proof, differently with respect to the definition of Colombeau real analytic function [23], we have that, generally speaking,  is not finite. For example, for $f=\delta$ at $c=0$, we have $\left| \frac{\delta _{\varepsilon }^{(n)}(x_{\varepsilon })}{n!}\right| =\left| \frac{\mu ^{(n)} (x_{\varepsilon })}{n!}b_{\varepsilon }^{n+1}\right| =\left| \frac{\mu ^{(n)}(x_{\varepsilon })}{n!}b_{\varepsilon } \right| \frac{1}{\left( b_{\varepsilon }^{-1}\right) ^{n}}\le \frac{{\bar{C}}b_{\varepsilon }}{(b_{\varepsilon }^{-1})^{n}}$, where $\left| \mu ^{(n)}(x_{\varepsilon })\right| \le \int \beta =:{\bar{C}}$ and hence $\frac{1}{R}=b$ which is an infinite number. Thereby, in the particular case when $\frac{1}{R}$ is finite, *f* is a Colombeau real analytic function in a neighborhood of *c*. Vice versa, any Colombeau real analytic function and any ordinary real analytic function are GRAF.

This characterization also yields the closure of GRAF with respect to inversion. We first recall that the local inverse function theorem holds for GSF, see [9]. Therefore, if  and at the point $x_{0}\in U$ the derivative $f'(x_{0})$ is invertible, we can find open neighborhoods of $x_{0}\in X\subseteq U$ and of $y_{0}:=f(x_{0})\in Y$ such that $f|_{X}:X\rightarrow Y$ is invertible,  and $f'(x)$ is invertible for all $x\in X$.

### Theorem 38

If $\sigma \le \rho ^{*}$ and we use notations and assumptions introduced above, then .

### Proof

For simplicity, set $g:=\left( f|_{X}\right) ^{-1}$ and $h(x):=\frac{1}{f'(x)}$ for all $x\in X$, so that $g'(y)=h[g(y)]$ for all $y\in Y$. From Corollary 32 and Theorem 26, we know that *h* is a GRAF. Therefore, Theorem 37 yields $\forall ^{0}\varepsilon \,\forall n\in \mathbb {N}:\ \left| h_{\varepsilon }^{(j)}(x_{\varepsilon })\right| \le C_{\varepsilon }\frac{j!}{R_{\varepsilon }^{j}}$ for all $[x_{\varepsilon }]\in B_{s}(x_{0})$ and suitable constants *s*, *C*, . For $[y_{\varepsilon }]\in f\left( B_{s}(x_{0})\right)$ (note that this is an open neighborhood of $y_{0}$ because *f* is an open map) and these $\varepsilon$, formula (1.15) of [20, Theorem 1.5.3] yields $\left| g_{\varepsilon }^{(j)}(y_{\varepsilon })\right| \le j!(-1)^{j-1}{1/2 \atopwithdelims ()j}\frac{(2C_{\varepsilon })^{j}}{R_{\varepsilon }^{j-1}}$ for all $j\in \mathbb {N}_{>0}$, and hence  once again by Theorem 37. $\square$

Since $\delta$ is a GRAF, in general the identity principle does not hold for GRAF. From our point of view this is a feature of GRAF because it allows to include as GRAF a large class of interesting generalized functions and hence pave the way to a more general related Cauchy-Kowalevski theorem. The following theorem clearly shows that the identity principle does not hold in our framework exactly because we are in a non-Archimedean setting: every interval is not connected in the sharp topology because the set of all the infinitesimals is a clopen set, see e.g. [8].

### Theorem 39

Let  be an open set and *f*, . Then the set

$$\begin{aligned} \mathcal {O}:=\text {{int}}\left\{ x\in U\mid f(x)=g(x)\right\} \end{aligned}$$

is clopen in the sharp topology.

### Proof

For simplicity, considering $f-g$, without loss of generality we can assume $g=0$. We only have to show that $\mathcal {O}$ is closed in *U*. Assume that *c* is in the closure of $\mathcal {O}$ in *U*, i.e.4.4We have to prove that $c\in \mathcal {O}$. We first note that for each ${\bar{c}}\in \mathcal {O}$, we have $B_{p}({\bar{c}})\subseteq \mathcal {O}$ for some  and hence

4.5 $$\begin{aligned} f({\bar{x}})=0\quad \forall {\bar{x}}\in B_{p}({\bar{c}}). \end{aligned}$$

Now, fix $n\in \mathbb {N}$ in order to prove that $f^{(n)}(c)=0$. From (4.4), for all  we can find ${\bar{c}}_{r}\in B_{r}(c)\cap \mathcal {O}$ such that $f^{(n)}({\bar{c}}_{r})=0$ from (4.5). From sharp continuity of $f^{(n)}$, we have $f^{(n)}(c)=\lim _{r\rightarrow 0^{+}}f^{(n)}({\bar{c}}_{r})=0$. Since  and $c\in U$, we can hence find $\sigma >0$ such that  for all $x\in B_{\sigma }(c)$, i.e. $c\in \mathcal {O}$. $\square$

For example, if $f=\delta$ and $g=0$, the setis clopen. Thereby, also  is clopen, and we haveIf we assume that all the derivatives of *f* are finite and the neighborhoods of Definition 30 are also finite, then we can repeat the previous proof considering only standard points $c\in \mathbb {R}$ and radii $r\in \mathbb {R}_{>0}$, obtaining the following sufficient condition:

### Theorem 40

Let  be an open set such that $U\cap \mathbb {R}$ is connected. Let *f*,  be such that $f|_{V\cap \mathbb {R}}=g|_{V\cap \mathbb {R}}$ for some nonempty subset $V\subseteq U$ such that $V\cap \mathbb {R}$ is open in the Fermat topology, i.e.

$$\begin{aligned} \forall x\in V\cap \mathbb {R}\,\exists r\in \mathbb {R}_{>0}:\ B_{r}(x)\subseteq V\cap \mathbb {R}. \end{aligned}$$

Finally, assume that all the following quantities are finite:

(i) The neighborhood length *s* in Definition 30 is finite for each $c\in U\cap \mathbb {R}$,
(ii) $\forall x\in U\,\forall n\in \mathbb {N}:\ f^{(n)}(x)$ and $g^{(n)}(x)$ are finite.

Then $$f|_{U\cap \mathbb {R}}=g|_{U\cap \mathbb {R}}$$.

### Proof

The proof proceeds exactly as in Theorem 39 but considering

$$\begin{aligned} \mathcal {O}:=\text {int}_{\text {F}}\left\{ x\in U\cap \mathbb {R}\mid f(x)=g(x)\right\} , \end{aligned}$$

where $\text {int}_{\text {F}}$ is the interior in the Fermat topology (i.e. the topology generated by the balls $B_{r}(c)$ for  and $r\in \mathbb {R}_{>0}$, see [8]). We have to note that assumption (ii) implies that all $f^{(n)}$ are continuous in this topology (see [11]). $\square$

For example, if $f\in \mathcal {C}^{\omega }(\mathbb {R})$ is an ordinary real analytic function and *K*,  are finite numbers, the GRAF , where  is the set of compactly supported points, satisfies the assumptions of the last theorem.

## Conclusions

Sometimes, e.g. in the study of PDE, the class of real analytic functions is described as a too rigid set of solutions. In spite of their good properties with respect to algebraic operations, composition, differentiation, integration, inversion, etc., this rigidity is essentially well represented by the identity principle that necessarily excludes e.g. solitons with compact support or interesting generalized functions. Thanks to Theorem 39, we can state that this rigidity is due to the banishing of non-Archimedean numbers from mathematical analysis. The use of hyperseries allows one to recover all these features including also interesting non trivial generalized functions and compactly supported functions. This paves the way for an interesting generalization of the Cauchy-Kowalevski theorem for GRAF that we intend to develop in a subsequent work. Its proof can be approached by trying a generalization of the classical method of majorants, or using the Picard-Lindel-f theorem for PDE with GSF and then using characterization Theorem 37 to show that the GSF solution is actually a GRAF.
